# Molecular Machines For The Control Of Transmembrane Transport

**DOI:** 10.1021/jacs.3c08877

**Published:** 2023-12-08

**Authors:** Toby G. Johnson, Matthew J. Langton

**Affiliations:** Department of Chemistry, Chemistry Research Laboratory, University of Oxford Mansfield Road, Oxford OX1 3TA United Kingdom

## Abstract

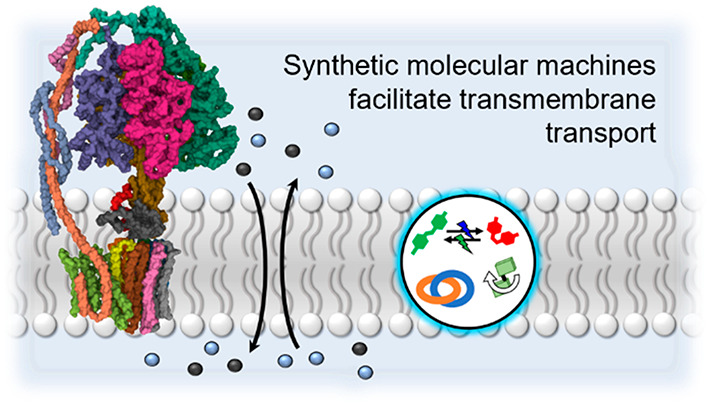

Nature embeds some of its molecular machinery, including ion pumps, within lipid bilayer membranes. This has inspired chemists to attempt to develop synthetic analogues to exploit membrane confinement and transmembrane potential gradients, much like their biological cousins. In this perspective, we outline the various strategies by which molecular machines—molecular systems in which a nanomechanical motion is exploited for function—have been designed to be incorporated within lipid membranes and utilized to mediate transmembrane ion transport. We survey molecular machines spanning both switches and motors, those that act as mobile carriers or that are anchored within the membrane, mechanically interlocked molecules, and examples that are activated in response to external stimuli.

## INTRODUCTION

Molecular machines lie at the heart of almost all biological processes, operating at length scales where random thermal fluctuations dominates their motion. This ubiquity has inspired chemists to strive toward developing artificial analogues, mimicking the roles of their natural cousins, or identifying entirely artificial functions. The initial developments in this field of Sauvage, Stoddart, and Feringa were recognized by the 2016 Nobel Prize in Chemistry.^1−3^ While numerous sophisticated systems have been reported in solution, due to the random tumbling of molecules, in most cases, the controlled nanomechanical motions of molecular machines are effectively randomized. The spatial organization of molecular machines, such as on a surface, is therefore of particular importance in order to extract useful work from the assembly. Nature immobilizes some of its molecular machinery within lipid bilayer membranes. For example, the membrane-bound ion pump ATP-ase transduces energy from ATP hydrolysis to pump protons across the cellular membrane, in a process accompanied by unidirectional rotation of the protein (or, in the reverse mode, utilizing the energy stored in the ion gradient to catalyze the chemical synthesis of ATP).^4^ The sophistication and out-of-equilibrium functions of this biological molecular machine have so far remained out of reach of synthetic systems. For an overview of the state of the art in solution phase research, including pumps, motors and chemically fuelled systems, as well as surface bound molecular machines, the reader is directed to a number of recent reviews.^5−8^

Artificial molecular machines in membranes have remained comparatively rare, and initially were primarily composed of modified biological machines such as those derived from nanopores.^9^ This field was last reviewed by Watson and Cockcroft in 2015, and in the following eight years, there has been a step-change in developing entirely artificial membrane confined molecular machines. Practically speaking, by incorporating organic molecular machine components into hydrophobic membranes, chemists have at their disposal a versatile method of interfacing lipophilic supramolecular chemistry with aqueous biological environments (noting that the vast majority of functional organic/inorganic molecular machines are not water soluble, limiting their application in biological contexts). Because of this, membrane chemistry is arguable one of the most promising areas in which we can expect to see useful applications of molecular machines through its impact on biology and medicine. Given the rapid recent progress in this regard, in this perspective, we aim to take stock of where the field is at, to categorize the various new mechanisms of ion transport based on the nanomechanical motions of synthetic molecular machines, and provide a perspective on where we envisage the next challenges and developments will be, in terms of both fundamental science and practical applications.

### Ion Transport by Synthetic Supramolecular Systems: Mechanistic Diversity

**Figure 1:**
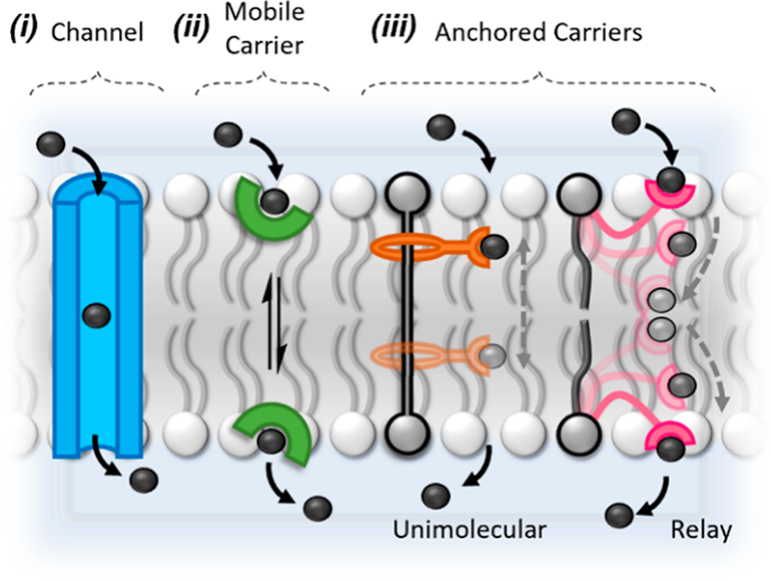
Mechanisms of transmembrane ion transport. Schematic of transmembrane ion channels (i), mobile carriers (ii), and anchored carriers (iii), which can be either unimolecular or relay-based systems.

In nature, ion transport is mediated primarily by transmembrane protein channels or sophisticated biomolecular machine ion pumps and, to a lesser extent, by mobile carrier (also referred to as ionophores). A wide range of synthetic ion channels and mobile carriers have emerged,^10,11^ including those with stimuli-responsive behavior.^12−14^ Channels provide a pore through which ions may flow down their concentration gradient (Figure 1.i) while mobile carriers shuttle ions across the membrane, via consecutive binding, translocation, and release steps (Figure 1.ii). A range of different intermolecular interactions have been employed for the binding of ions to promote transport; we direct the reader to recent reviews on metal-organic-based transporters,^15,16^ hydrogen bonding systems^17,18^ and transporters utilizing sigma-hole interactions.^19,20^ For reviews concerning the methods developed for the study of ion transport, we direct the reader to the following.^21−24^

Recently, new abiotic mechanisms of transport that exploit the nanomechanical motions of molecular machines have emerged, such as relays, swings, and shuttles, vide infra. These novel mechanisms of transport typically rely on an anchoring group to provide some degree of immobilization of the transporter in the bilayer, while a mobile component capable of large amplitude molecular motion (e.g., macrocycle component of a rotaxane or carrier arm of a relay) facilitates transport (Figure 1.iii). We propose to define such ion transport mechanisms as “*anchored carriers*”, because they incorporate features of channels (anchored to the membrane and immobile) and carriers (requiring motion of a receptor between both membrane interfaces). The work of Gokel and co-workers in the 1990s provides early inspiration for anchored carrier systems, in which channels based on crown ethers connected by flexible linkers were anchored within the membrane.^25,26^ While these systems operated via a channel mechanism, the structural characteristics of these early transporters provide inspiration for anchored carrier systems.

In this perspective, we adopt a broad definition of molecular machine, namely, a molecule or assembly of molecules in which nanomechanical motion is exploited for a function. Within this broad category, we can further subdivide between switches and motors: the former cannot do work, while the latter are able to.^5^ From a more molecular perspective, a molecular switch is a species capable of reversible and repeated interconversion between two states via some degree of nanomechanical molecular motion, which in the context of ion transport has commonly been utilized to switch activity on and off. Molecular motors, on the other hand, are able to do work by transducing energy from photons or a chemical fuel. These include, but are not limited to, molecular rotors, which are molecules that undergo unidirectional rotation under photoirradiation, and have been exploited to permeabilize membranes or enhance the activity of ion transporters. A key feature of molecular machines is the stimuli-responsive control over motion, with pH changes, temperature, ligand binding, redox chemistry, light, and other stimuli having been reported.^27^ We highlight these systems within this perspective, alongside interlocked ion transporters, which are themselves an important class of molecules within the rapidly expanding field of molecular machine development.

## TRANSMEMBRANE CHANNELS

Transmembrane protein channels and pumps are the predominant means of regulating ion gradients in biology. Stimuli-responsive opening and closing of gated-channels controls the function of many biological processes within the cell, and engineering of photoresponsive biological machinery in the form of cellular membrane channels is the basis of the field of optogenetics.^28^ Many sophisticated examples of regulating protein channels using synthetic molecular photoswitches have been reported, but are beyond the scope of this perspective and have been reviewed elsewhere.^28−30^ We focus our discussion on wholly synthetic examples and direct the reader to the reviews cited for more information about these topics.

### Mechanically Interlocked Ion Channels

**Figure 2:**
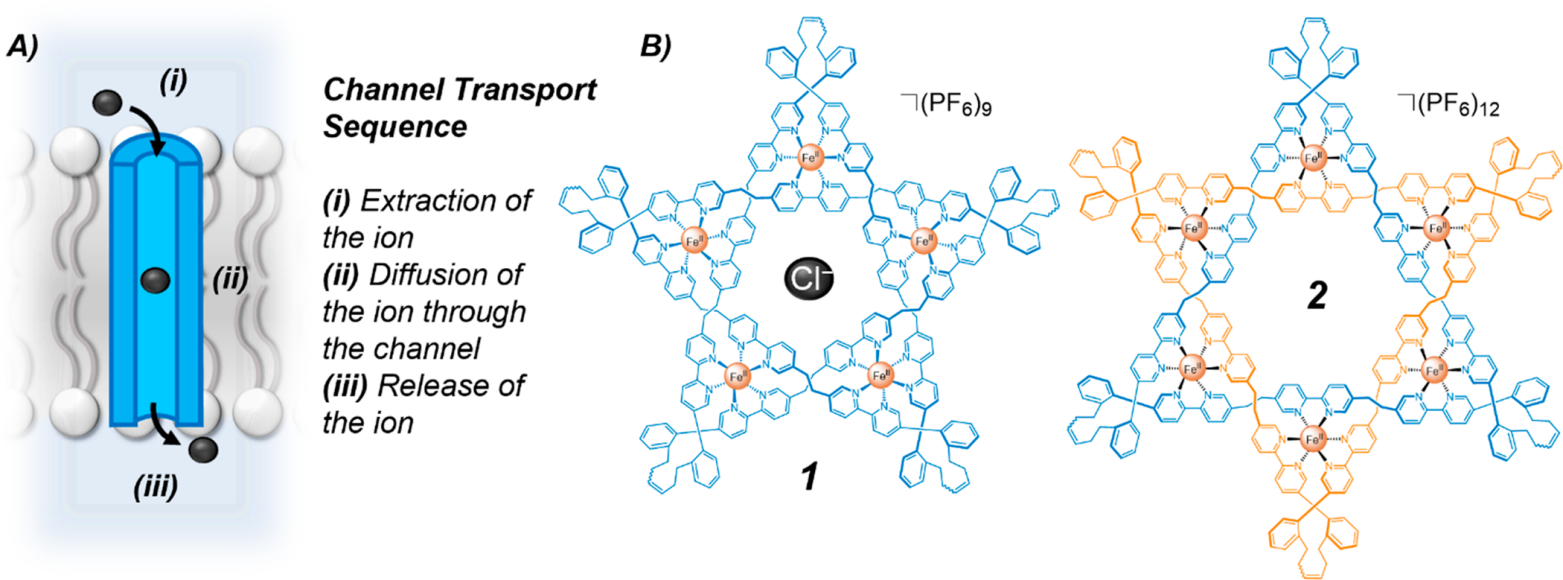
Interlocked ion channels. A. Schematic of the transmembrane channel transport sequence. B. Pentafoil knot **1** and Star of David [2]catenane **2** chloride ion channels.^31^

In 2020, Leigh and co-workers demonstrated that the complex mechanically interlocked topology of a Star of David [2]catenane was essential to its transport activity.^31^ Two metalated interlocked structures, (Fe^II^)_5_-coordinated pentafoil knot **1** and (Fe^II^)_6_-coordinated Star of David [2]catenane **2**, were shown to mediate anion transport when incorporated into large unilamellar vesicle (LUV) membranes (Figure 2). The Star of David channel **2** displayed more than 50-fold greater activity compared to knot **1**, which was attributed to the larger cavity size of the Star of David. The Fe(II) cations in the complex were shown to be essential for activity—the demetalated [2]catenane was completely inactive in the 8-hydroxy-1,3,6-pyrenetrisulfonic acid (HPTS) assay. The cations act to both rigidify the channel structure and enhance anion binding. The hexameric cyclic helicate precursor to **2** was found to be inactive, demonstrating that the conformational constraints imposed by interlocking were crucial to the transport activity. While the interlocked topology was shown to be essential for activity, the structure undergoes no nanomechanical motion (and thus cannot be described as a molecular machine). However, given that mechanically interlocked molecules are the basis of a huge array of molecular machine type systems in solution, future developments of such knotted architectures may have the potential for dynamic molecular machine activity within membranes.

**Figure 3:**
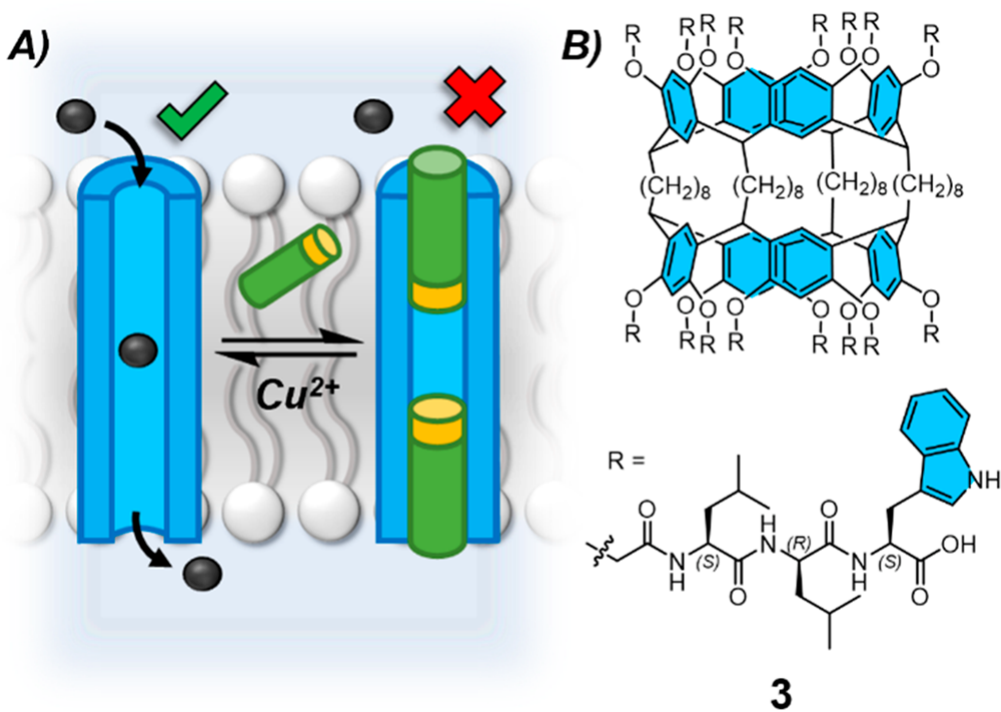
A. Reversible ligand-gated formation of pseudorotaxane architectures controls ion channel activity. B. Peptide-appended bis-resorcinarene unimolecular channel (**3**) with reversible ligand-gated cation transport activity.^33^

Formation of a pseudorotaxane interlocked structure, in which a blocking ligand threads through an ion channel, has been demonstrated as an approach to reversibly gate the transport activity of channel **3** (Figure 3).^32−34^ The peptide-appended bis-resorcinarene **3** afforded regular square-like signals in black lipid membrane (BLM) single channel conductance experiments, indicative of transmembrane channel formation. Straight chain alkyl amines (octyl to octadecyl) inhibited the unimolecular channel cation transport activity, where longer alkyl chains demonstrated an improved ability to block channel **3**. The blocking ligands could be removed from the pseudorotaxane type host–guest complex by Cu^2+^ cations which sequestered the amines and restored transport activity.

Pelta and co-workers have also reported α-cyclodextrin nanotube channels,^35−37^ which were synthesized via Harada’s rotaxane templated synthesis method^38^ via threading of α-cyclodextrins onto a polymer axle. Employing the interlocked structure to favor the coupling of neighboring α-cyclodextrins gave threaded nanotube channels. These threaded channels showed diminished transport activity, while hydrolysis and dethreading yielded efficient ion channels.

### Molecular Switch-Based Transmembrane Channels

**Figure 4:**
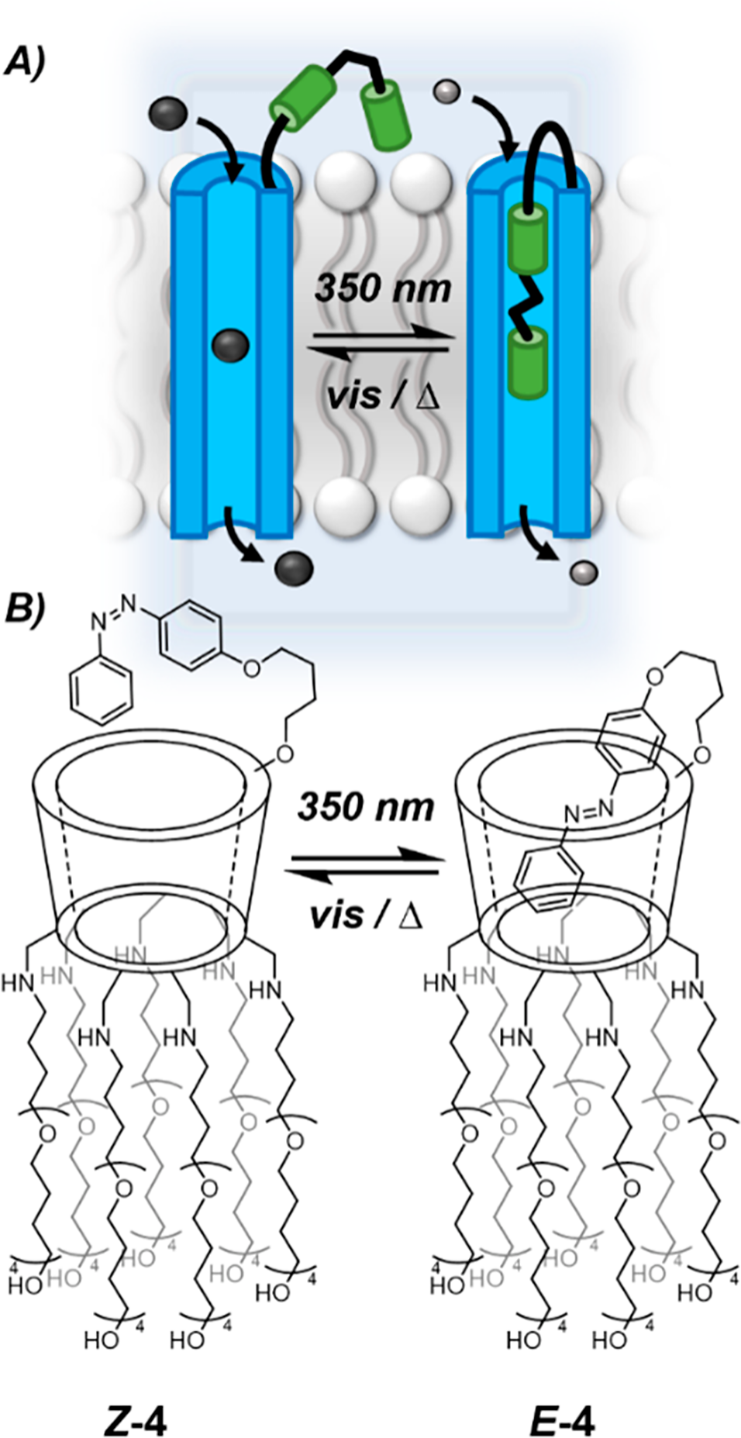
A. Reversible photocontrol of channel pore size controls ion channel selectivity. B. Photoswitching of **4** via pseudorotaxane formation controls ion selectivity of this β-cyclodextrin based channel.^40^

The same principal of regulating transport through reversible blocking of a channel pore, as a pseudorotaxane or inclusion complex, has been demonstrated with azobenzene appended ion channels.^39,40^ Gin and co-workers reported a β-cyclodextrin-based ion channel that displayed switchable ion transport selectivity in response to the configuration of the appended azobenzene switch (Figure 4).^40^ When in the *E*-configuration, the azobenzene forms a self-inclusion complex ***E*****-4**, partially blocking the pore and only allowing smaller sodium cations to be transported. In contrast, *Z*-isomer ***Z*****-4** does not adopt the threaded β-cyclodextrin conformation to block the pore, enabling the larger chloride anions to be transported. Characterization of the inclusion complex within the bilayer was challenging; however, the demonstration of a change in activity in response to conformational switching supports the likely structural blocking of the pore.

**Figure 5:**
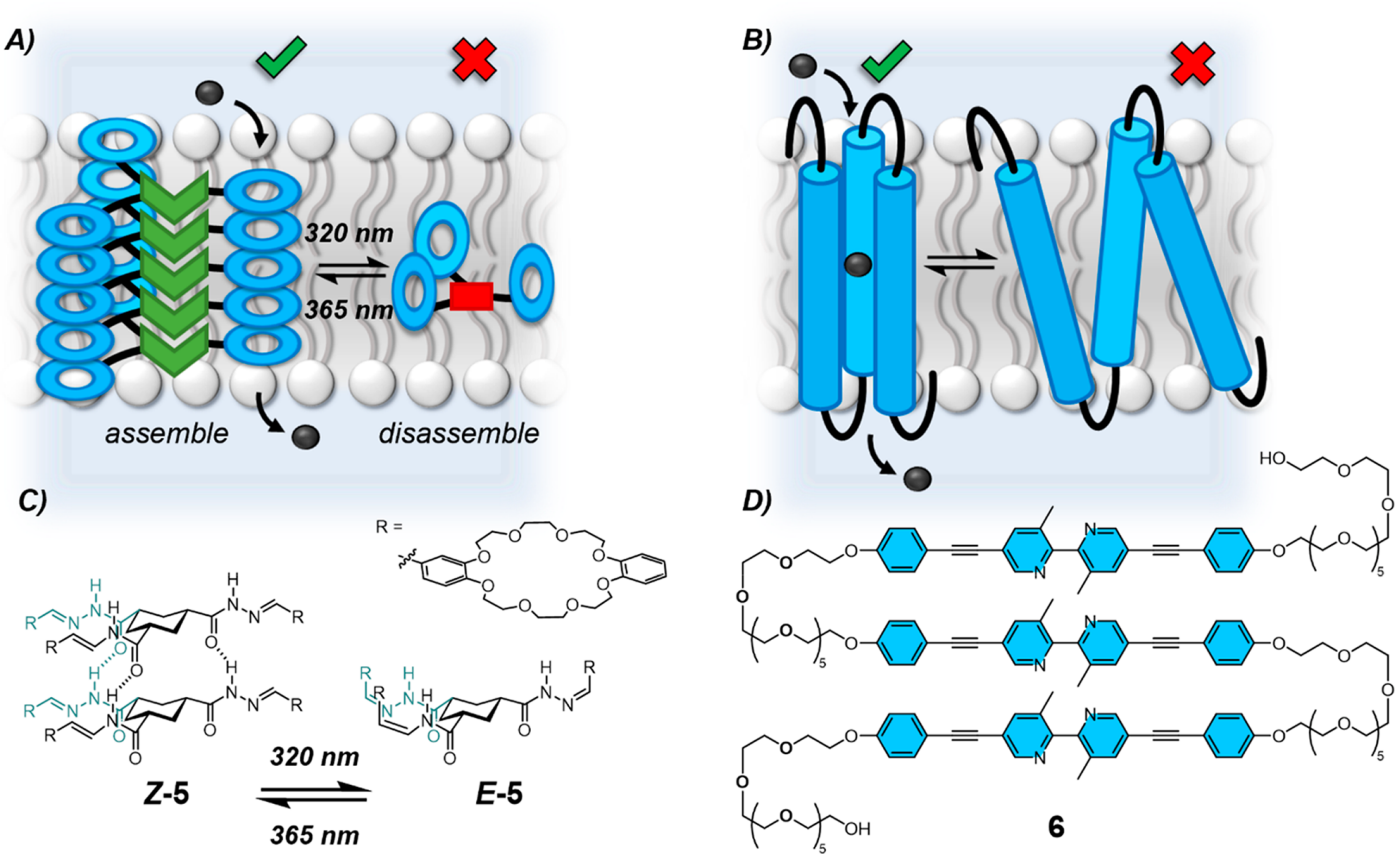
A. Photoswitchable control of a self-assembly channel. B. Reversible switching of conformation in response to membrane tension regulates ion channel activity. C. Acylhydrazone-linked crown ether triad **5** self-assembles into hydrogen-bonded stacks of ***Z*****-5**, but cannot when in the *E*-configuration.^41^ D. The BPBP trimer **6** serves as a mechanosensitive K^+^ ion channel.^42^

An alternative strategy to control transport activity with molecular switches has been to control the molecular self-assembly of multicomponent channels within the bilayer. Reversible photocontrol of azobenzene^43,44^ and acylhydrazone^41^ photoswitches has been demonstrated for the regulation of cation transport in model membranes. The self-assembly of stacks of the acylhydrazone-linked crown ether triad **5** could be controlled in the bilayer to turn transport on and off with light (Figure 5).^41^ In the *Z*-configuration, intermolecular hydrogen bonding held the stacks together and resulted in efficient transport of cations through dibenzo-24-crown-8 channels. Irradiation with 320 nm light photoswitched the acylhydrazone to the *E*-configuration and resulted in disassembly of the channel with a concomitant loss of transport activity, while 365 nm photoirradiation regenerated the *Z*-configuration and reassembly restored channel activity.

Mechanical stimulation has been applied to the reversible conformational switching of a multipass transmembrane channel.^42^ Mechano-sensitive ion channels are common in nature; however, synthetic analogues are extremely rare.^45^ Kinbara and co-workers have reported a transmembrane trimer **6** consisting of repeating oligo(ethylene glycol) chains and 3,3′-dimethyl-5,5′-bis(phenylethynyl)-2,2′-bipyridine (BPBP) units assembled as a multipass channel in the lipid bilayer (Figure 5).^42^ The hydrophobic BPBP subunits span the hydrophobic portion of the membrane, adopting either an expanded or contracted conformation in the bilayer. With a contracted membrane, the BPBP units are forced to compact, forming a transient pore mediating K^+^ ion transport. However, with an expanding tension, the pore collapsed, resulting in loss of transport activity. The conformation of this channel could be controlled with membrane tension, with reversible control, thus acting as a molecular switch. Importantly, this conformational switching was the direct result of assembly within the bilayer, demonstrating the efficacy of the bilayer environment to organize molecules into conformations which display more complex functions than would be achievable in bulk solution, and as a means to transduce mechanical force into molecular level effects.

β-barrel ion channels reported by Matile and co-workers^46,47^ have also been shown to display conformational switching within the lipid bilayer, to regulate the flow of ions through the pore.^48−50^ In these systems, dialkoxynaphthalene ligand binding to naphthalene diimide-derived rigid-rod scaffolds leads to a global conformational change and opening of the channel. This is triggered by intercalation of the electron-rich dialkoxynaphthalene ligands between the electron-deficient naphthalene diimides to form a π-stack architecture which stabilizes the channel.

### Molecular-Motor-Based Transmembrane Channels

**Figure 6:**
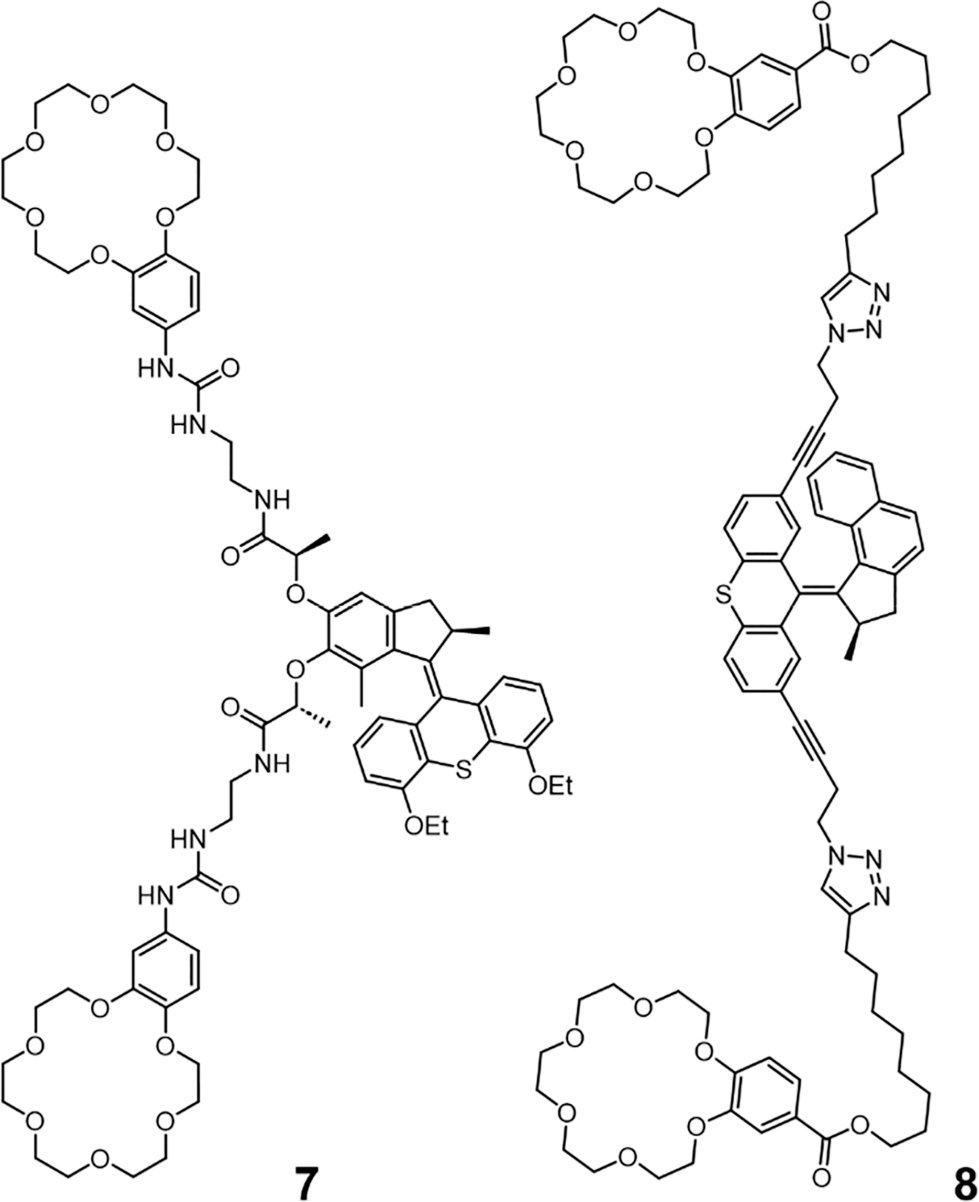
Rotation of the molecular motor conjugated to ion channels enhances transport activity. Molecular motor conjugated ion channels reported by Barboiu and Giuseppone (**7**)^51^ and Qu and Bao (**8**),^52^ which display enhanced transport activity under constant irradiation that induces unidirectional rotation.

Very recently, molecular motors have been incorporated into the structure of ion channels, to regulate the transport activity with controlled unidirectional rotation of Feringa type light-driven rotary motors (Figure 6).^51,52^ Two such systems have been reported by Barboiu and Giuseppone (**7**), as well as Qu and Bao (**8**), which incorporated benzo-18-crown-6 cation receptor units to elicit potassium-cation-selective ion channels. Urea motifs were integrated into **7** to promote the self-assembly of the channel structure in the membrane. Transport activity experiments in HPTS-loaded egg yolk phosphatidylcholine (EYPC) LUVs (100 nm) found the channel to be cation-selective with the following relative rates of transport: Rb^+^ > K^+^ > Na^+^. BLM single channel conductance measurements provided unequivocal evidence of a channel transport mechanism. Under constant 365 nm irradiation, the transport activity was significantly enhanced in both LUV and BLM studies, where covalently coupling the motor to the channel was shown to be essential to the enhanced transport activity. This enhancement was postulated to be due to the local deformations in microphase and increased fluidity due to local heating through energy dissipation.^53^

Interestingly, studies with rotor **8**, reported by Qu, Bao and co-workers, demonstrated the same mechanism of light-driven motor rotation enhanced transport activity. The channel was K^+^-selective and displayed enhanced activity with 365 nm irradiation. A calcein leakage assay demonstrated that even under UV irradiation, **8** did not cause appreciable membrane disruption or nonspecific leakage. Further studies of **8** in cellular membranes showed the same photodependent transport activity for K^+^, which induced cell apoptosis in HeLa cells demonstrating the potential of these systems for cancer therapy. This new mechanism of regulating ion transport through the continuous out-of-equilibrium rotation of a molecular motor represents a major step toward the sophisticated operation of biological machinery such as ATP synthase. These systems do not yet demonstrate active transport of ions against a concentration gradient, but the stimuli-responsive regulation of activity with molecular motor motion is a significant step forward in the complexity of membrane-bound artificial molecular machines.

## MOBILE CARRIERS

A wide variety of synthetic mobile carriers have been reported, utilizing a range of intermolecular interactions to selectively transport a desired ion, with an increasing emphasis on incorporating stimuli-responsive behavior to exert control over their activity (see the Introduction for references to a range of reviews on this topic). Anion carriers operate via consecutive ion binding, membrane translocation, and ion release steps, and are typically characterized by anion binding-limited transport activity,^54,55^ although some rare examples of translocation-limited mobile carriers have been reported.^56,57^ For the development of stimuli-responsive mobile carriers, including those based on molecular machines, it is therefore important to consider the impact of changing receptor interactions on the ion binding step as well as carrier geometry and lipophilicity, in order to influence the translocation rate of the ion–mobile carrier complex. Recently, the interplay of binding and translocation kinetics have also been highlighted to be essential to the selectivity of mobile carriers.^58^

### Mechanically Interlocked Mobile Carriers

Mechanically interlocked structures, both naturally derived and synthetically obtained, are beginning to gain greater traction in biomedical applications.^59−61^ While numerous examples of synthetic mobile carriers have been reported, which employ a variety of intermolecular interactions to facilitate transmembrane transport, those with a mechanically interlocked architecture remain very rare.

**Figure 7:**
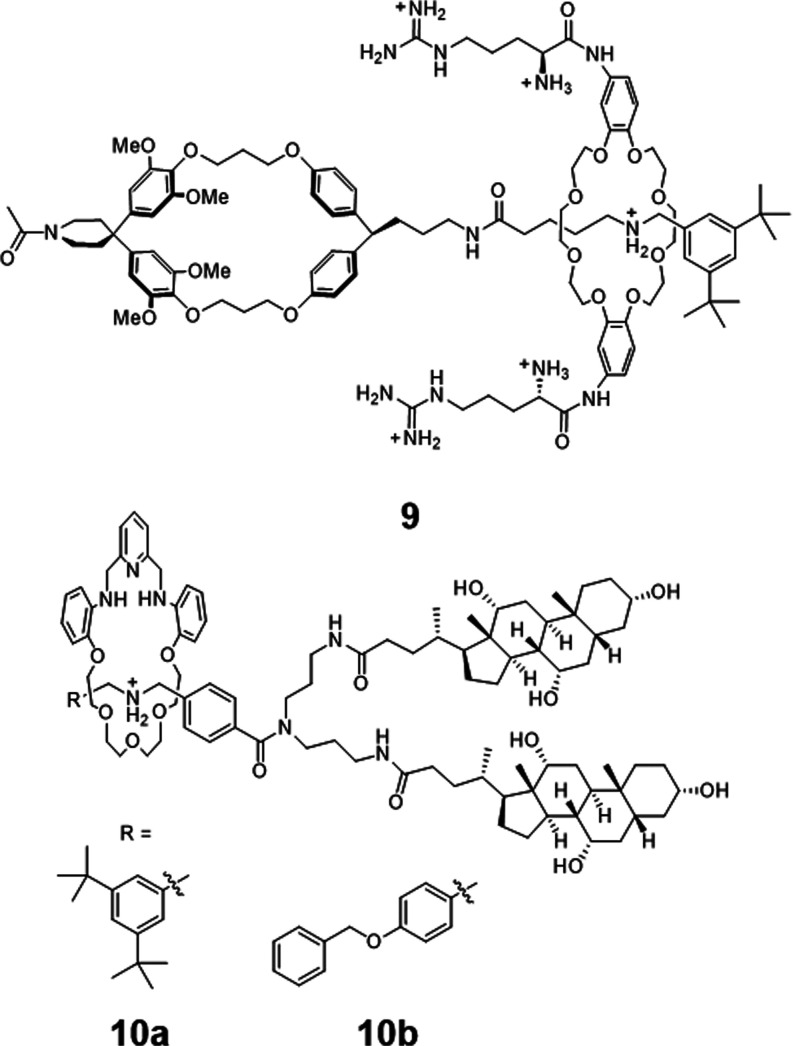
Interlocked molecular transporters via noncovalent recognition. Top: Smithrud’s cyclophane [2]rotaxane peptide transporter **9**.^62^ Bottom: Schmitzer’s umbrella rotaxane (**10a**) and pseudorotaxane (**10b**) Cl^–^ mobile carriers.^70,71^

The first example of an interlocked mobile carrier was reported by Smithrud and co-workers, who utilized [2]rotaxane **9** to facilitate the transport of fluorophores across cellular membranes (Figure 7).^62^ Rotaxane-mediated transport of dianionic fluorescein and fluorescein–peptide conjugates across cellular membranes enabled visualization of the cargo localization within COS-7 cells by fluorescence microscopy. Interestingly, in these systems the shuttling motion of the [2]rotaxanes was suggested to be key to transport activity:^63^ control experiments in which the secondary amine on the [2]rotaxane axle was acetylated revealed inhibited shuttling motion and reduced binding affinity for fluorescein in aqueous mediate relative to the dynamic [2]rotaxane, while binding in CHCl_3_ was unaffected. The ability to adapt the conformation to maintain a high binding affinity for the molecular cargo was critical in these systems, as the polarity of the bilayer environment changes significantly during the transport process. To confirm **9** acted as a mobile carrier which passively diffused across the cellular membrane, experiments were conducted at reduced temperature and with ATP-depleted COS-7 cells.^64^ Under these conditions, the transport activity was only moderately reduced, confirming that endocytosis was not the major pathway to fluorescein internalization. The cyclophane [2]rotaxane **9** was subsequently investigated for the transport of a range of fluorescein–peptide conjugates of various polarity in COS-7 and ES-2 cells as well as in bulk transport U-tube experiments.^64^ Studies on related [2]rotaxane derivatives have shown that an elongated polyethylene glycol axle improved aqueous solubility and hence transport activity, while a carboxylic acid or guanidine-functionalized macrocycle tuned transport selectivity for cationic peptide over negative charged or apolar peptide cargos.^65,66^ The same group have reported benzo-crown ether-appended [2]rotaxanes which displayed anticancer activity as a result of Ca^2+^ cation transport.^67,68^ A rhodamine B-derivatized [2]rotaxane allowed visualization of the membrane localization of this transporter, which facilitated the intracellular delivery of the anticancer drug doxorubicin for enhanced cytotoxic activity.^69^

Schmitzer and colleagues reported [2]rotaxane **10a** which facilitated Cl^–^ transport via a mobile carrier mechanism across a model lipid membrane.^70^ The rotaxane carrier was reported to have an EC_50_ value (concentration required to achieve half-maximal activity) of 1.56 mol % with resepct to lipid in EYPC vesicles, as determined using the chloride-sensitive fluorophore lucigenin encapsulated inside. Large amplitude conformational change resembling an umbrella motion was necessary for chloride transport in these systems as the structure translocated across the bilayer. A subsequent study found that self-assembly of pseudorotaxane **10b** within the bilayer served as a ligand-gated means of regulating transport activity.^71^ The unthreaded axle was found to be more active than the pseudorotaxane complex **10b**, where addition of the macrocycle component arrested transport.

**Figure 8:**
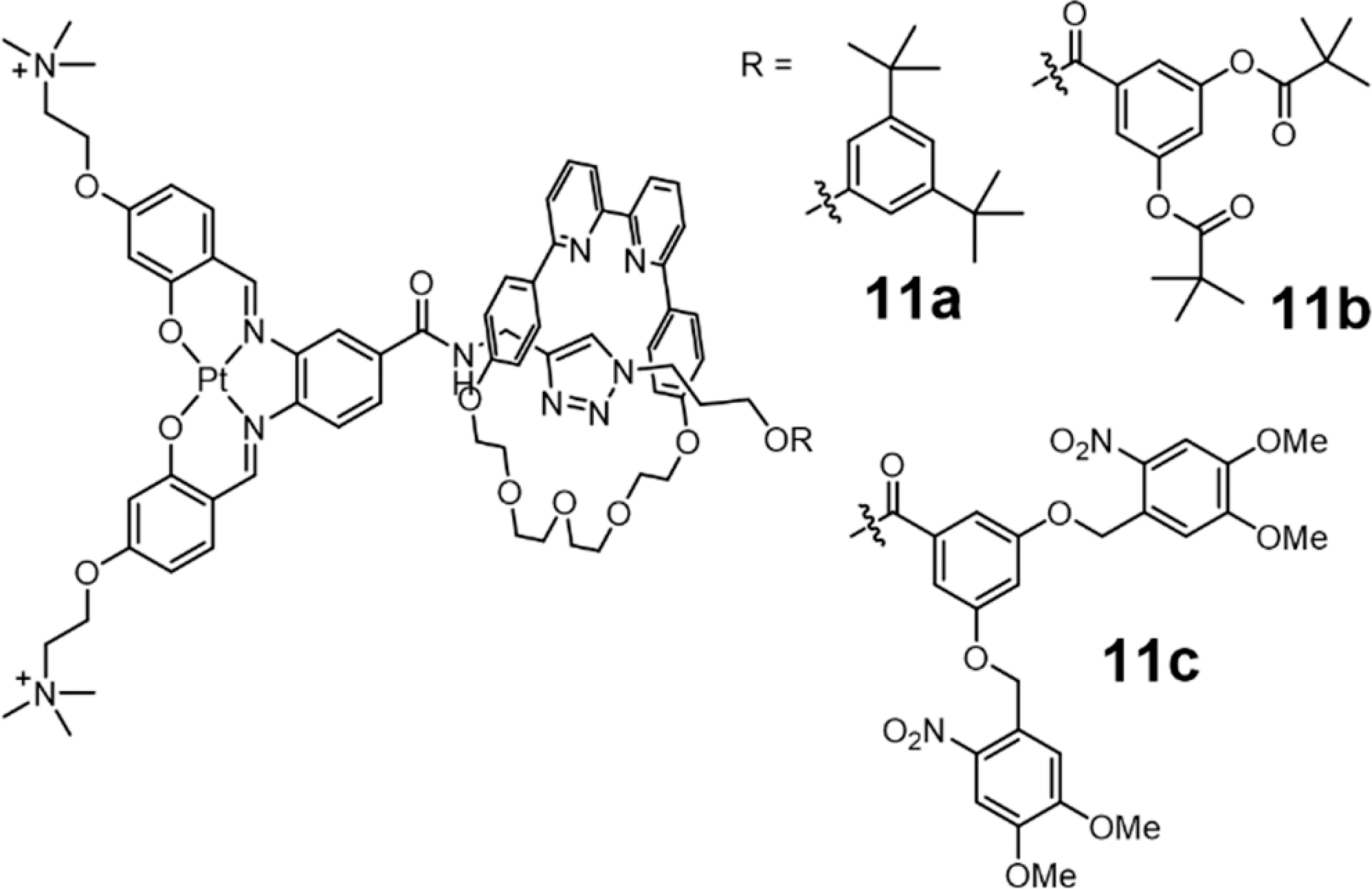
Interlocked molecular transporters using covalent cargo attachment. The [2]rotaxane architecture acts to improve cellular delivery of the cytotoxic Pt^II^-axle, while enzyme- (**11b**) and photo- (**11c**) responsive stoppers enable spatiotemporal control of activity.^77^

Another strategy for the transport of impermeable molecules into cells has been to covalently link the cargo as part of a rotaxane architecture,^72−76^ in which the interlocked structure improves membrane permeability and cellular uptake compared to the individual components. This strategy is, however, limited by the fact that interlocked structures must break down within the cell to release the cargo in an irreversible process. [2]Rotaxane **10a** (Figure 7), in addition to its noncovalent Cl^–^ transport capability, was also shown to be able to deliver macrocyclic cargo into EYPC vesicles via covalent attachment.^70^ In this work, the authors demonstrated the enzyme-mediated cleavage of amide bonds on the axle of **10a** by the protease α-chymotrypsin encapsulated within the vesicle lumen to release the macrocycle cargo. The same strategy was demonstrated with [2]rotaxane Pt prodrug **11** in U2OS osteosarcoma cells (Figure 8), which showed enhanced cell permeability and reduced cytotoxicity compared to the parent noninterlocked metallodrug.^77^ Three interlocked prodrugs were developed including noncleavable **11a** to serve as a control, which had little background cytotoxicity. Protease enzyme responsive **11b** and photoresponsive derivative **11c** enabled spatiotemporal control over the dethreading of the macrocycle, to reveal the active G-quadruplex DNA binder Pt^II^-axle which was responsible for cytotoxicity.

Recently, Langton, Beer, and co-workers reported the first catenane ion carriers, in which halide-selective binding via halogen bonds translated into high, therapeutically relevant Cl^–^ > OH^–^/NO_3_^–^ selectivity in anion transport studies.^78^ The activity of the interlocked [2]catenanes was improved > 3-fold relative to the most active macrocyclic component, despite the increase in molecular size, and demonstrates the potential for mechanically interlocked transporters which show improved activity and selectivity relative to their topologically simple components.

The interlocked systems described above have employed the mechanical bond to improve the analyte binding strength and membrane permeability, but these examples do not yet constitute true molecular machines. We anticipate that the future of interlocked mobile carriers likely will include transporters displaying enhanced selectivity resulting from the mechanical bond effect, activity dependent on the co-conformational motion of the interlocked components, and out-of-equilibrium ion pumps. Indeed, Stoddart and colleagues have already postulated that the incorporation of pseudorotaxane molecular pumps into lipid bilayers would enable the active transport of molecular cargo across membranes.^79^

### Mobile Ion Carriers Derived from Molecular Switches

Molecular switches have been incorporated into mobile carriers to enable stimuli-responsive transport activity. Typically, the molecular switching is utilized to control the proximity of two binding groups to improve the binding affinity and analyte encapsulation in the active state. Distinct from irreversible systems which allow the stimuli-responsive initiation or inhibition of transport activity, molecular switches facilitate fully reversible activation and deactivation of ion transport activity.^14^ While many different molecular switch architectures have been developed,^80^ the majority of photoswitchable mobile carriers reported to date have incorporated azobenzenes.^81−84^ Shinkai et al. initially established the application of molecular switches to control ion extraction between two liquid phases.^85^ Mobile carriers which transport cations across lipid bilayers have also been reported based on spiropyran,^86^ a bis-anthracene couple,^87^ and azobenzene.^84^

**Figure 9:**
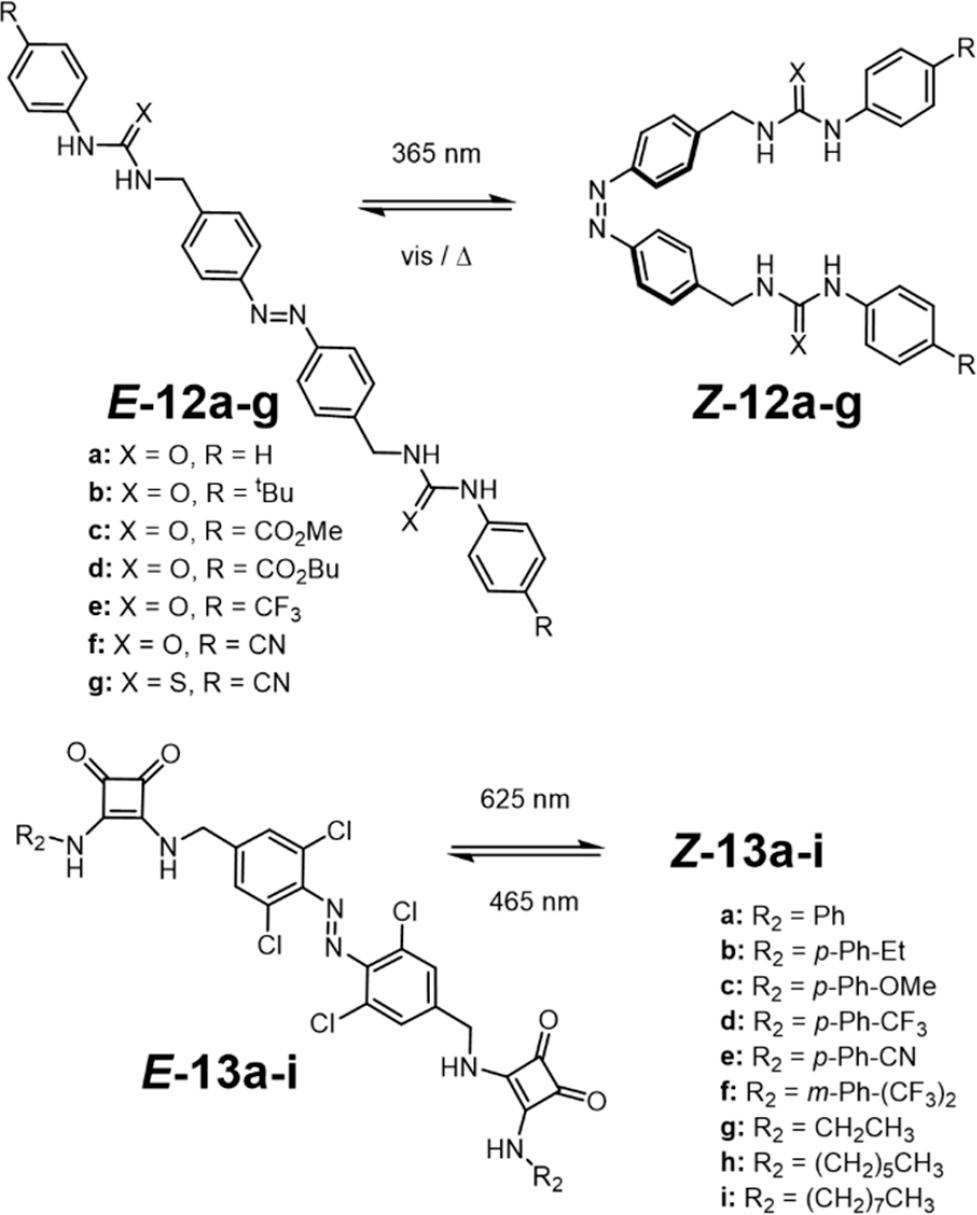
Photoswitchable mobile carriers. Bis-(thio)urea azobenzene (**12**)^88^ and red-shifted bis-squaramide tetra-chloroazobenzene (**13**) mobile carriers.^82^

In 2014, Jeong and co-workers were the first to demonstrate that anion transport activity in lipid bilayer membranes could be regulated through the use of a molecular switch to modulate the geometry of the mobile carriers **12a–g**.^88^ Their bis(thio)urea appended azobenzene transporter **12** could be photoswitched between a compact active *Z*-isomer and an open shape inactive *E*-isomer. In the *Z*-isomer, the receptor units are brought into close proximity to cooperatively bind a Cl^–^ anion, with binding constants found to be an order of magnitude greater compared to those of the corresponding open *E*-isomer. These transporters were also investigated within FRT cells, where Cl^–^ transport was visualized with the halide-responsive yellow fluorescent protein (YFP) expressed within the cells. The same activity trend in model membranes was seen across the cellular membrane, where the *Z*-isomer of the mobile carrier was more active than the *E*-isomer. However, reversible in situ switching of transport activity was not achieved in this system. Langton and co-workers subsequently utilized red-shifted tetra-*ortho*-chloroazobenzenes functionalized with squaramide anion binding groups **13a–i** (Figure 9) to facilitate reversible visible light (red and blue) responsive control of transport activity in a fully reversible, in situ fashion.^82,83^ Hydrazone switches have also been incorporated into anion transporters, which rely on different stimuli to switch between each state.^89,90^ Photoirradiation switches the hydrazone to a closed off state, while acid isomerizes the switch back to the active form.

**Figure 10:**
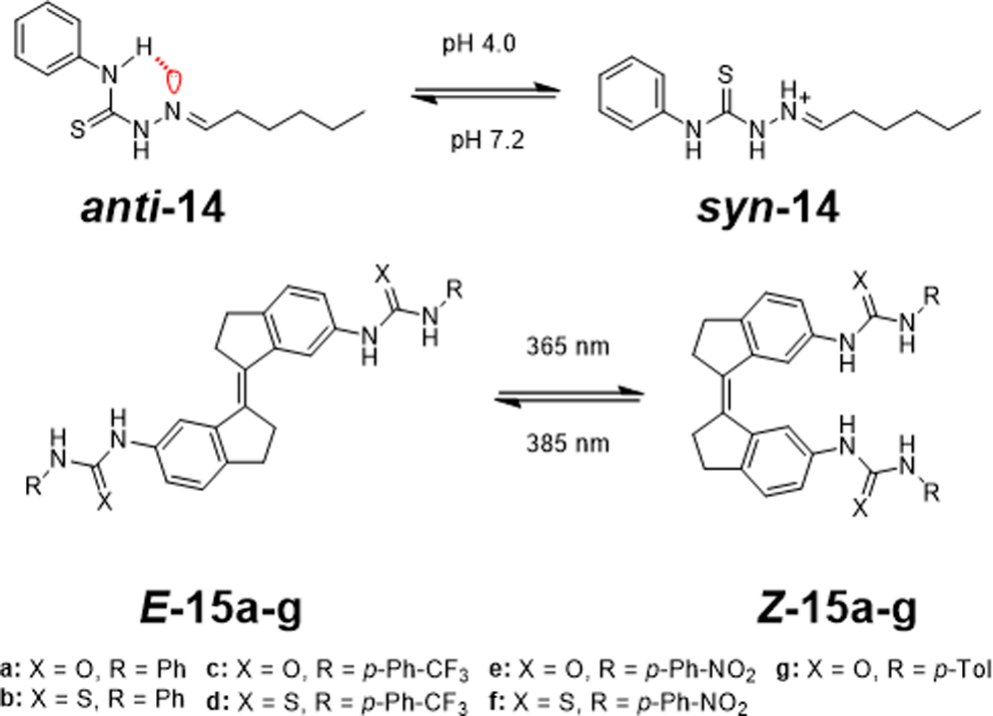
Selective mobile carriers based on molecular switches. pH-Responsive phenyl thiosemicarbazone H^+^/Cl^–^-selective symporter **14**(93) and stiff-stilbene photoresponsive Cl^–^-selective electrogenic transporter **15**.^95^

Beyond systems demonstrating stimuli-responsive switching between inactive and active states, those that also display high selectivity for a specific ion are highly desirable. pH-Regulated molecular switches, which change conformation upon protonation, have been incorporated within mobile carrier design^91^ to give highly selective transporters.^92−94^ Gale and co-workers reported a phenylthiosemicarbazone molecular switch **14** which facilitated H^+^/Cl^–^ symport exclusively in acidic conditions and diminished transport at neutral pH (Figure 10).^93^ Under neutral conditions, the intramolecular H-bond locks the structure into an *anti*-conformation with reduced anion binding affinity. In acidic conditions, the structure is protonated and adopts the *syn*-conformation, where both thiourea NH hydrogen bond donors are able to participate in anion binding. This pH-responsive molecular switch regulated transport activity and ensured highly selective cotransport of H^+^ and Cl^–^ ions, because only the ***syn*-14** HCl complex was neutral and therefore membrane-permeable. More recently, Wezenberg and co-workers reported stiff-stilbene-based photoswitchable anion mobile carriers.^95,96^ Bis(thio)urea derivative **15a–g** displayed photomodulated and selective Cl^–^ transport in 1-palmitoyl-2-oleoyl-*sn*-glycero-3-phosphocholine (POPC) vesicles.^95^ The *Z*-isomers of **15a–g** were found to have significantly improved transport activities compared to the respective *E*-isomers, with carrier **15e** displaying a 568-fold difference in activity between the isomers. The *Z*-isomers were highly selective for Cl^–^, functioning as electrogenic uniporters, and were able to photomodulate a membrane potential.

Recently, the photoregulated transport of oligoarginine peptides across model and cellular membranes was reported.^97^ A sulfonatocalix[4]arene receptor unit served to bind the cationic peptides, while a pendant azobenzene molecular switch modulated the lipophilicity of the transporter, which regulated the translocation step in the transmembrane transport process. In the *Z*-configuration, the azobenzene was more polar, and therefore transport was inhibited. Irradiation with 500 nm light isomerized the azobenzene to the more hydrophobic *E*-configuration, which promoted peptide transport.

## ANCHORED ION CARRIERS

In recent years, the scope of transporters has been broadened beyond the archetypal classification of either a mobile carrier or membrane-spanning channel.^9,98^ These new transporters can be described as “*anchored ion carriers*”: operating via molecular machine-inspired nanomechanical motion within the bilayer, but without the entire construct translocating through the membrane (as for mobile carriers). These are entirely abiotic mechanisms, distinct from their biological counterparts. Two clear classes of anchored carriers have emerged, namely unimolecular anchored transporters (where a single binding group facilitates the transport of the analyte through the entirety of the membrane) and relay transporters (where at least two binding groups are required to mediate the passing of the analyte between leaflets of the bilayer via an exchange step to enable transport).

### Unimolecular Anchored Transporters

**Figure 11:**
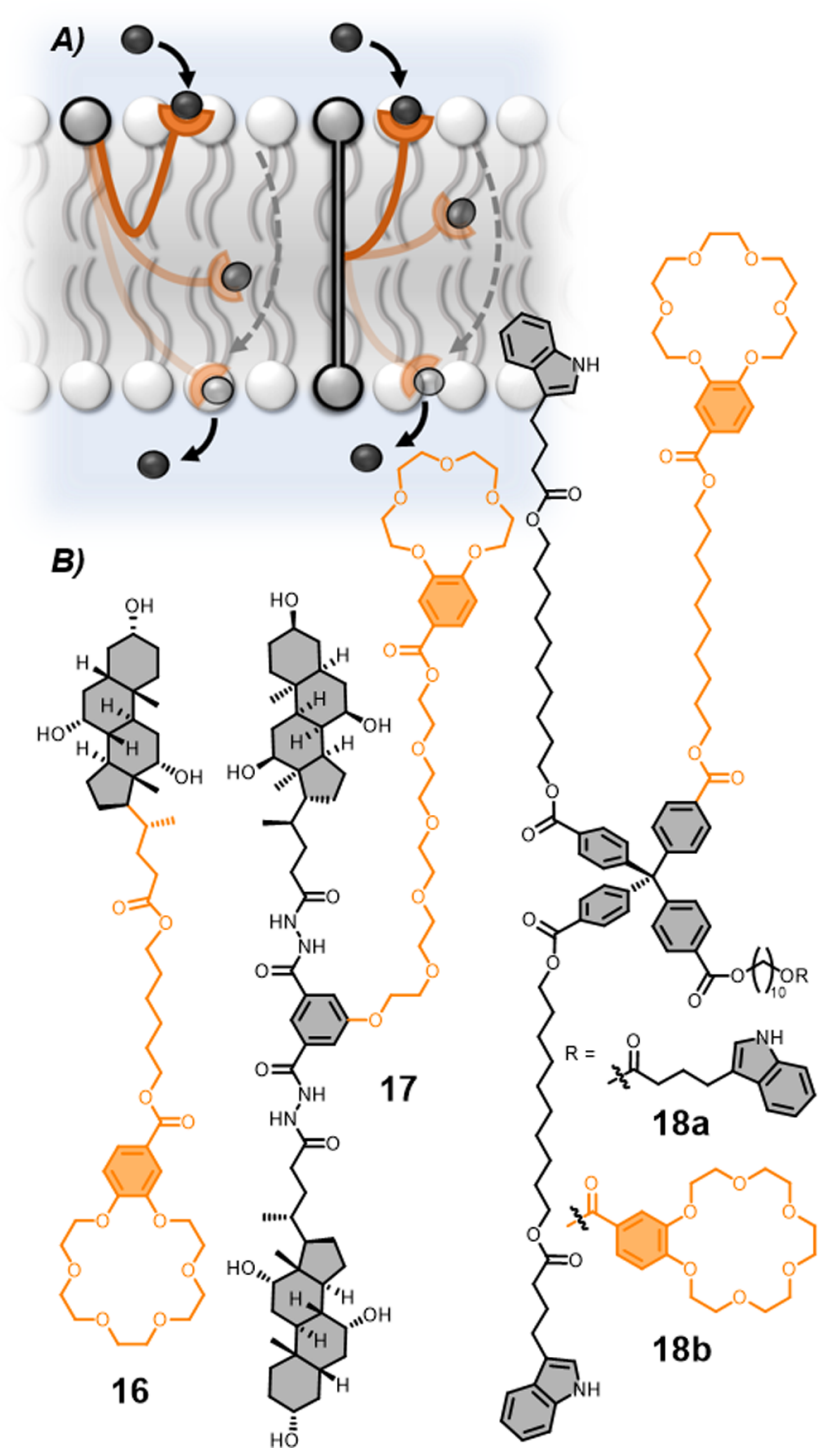
Unimolecular anchored carriers. A. Schematic of the unimolecular anchored carrier transport mechanism. B. Structures of monocholesterol anchored **16**,^99^ bis-cholesterol anchored **17**,^100^ and tris- and bis-indolyl anchored **18a–b**.^101^

A number of systems that act as unimolecular anchored transporters have been reported in recent years under a plethora of different names, which fall under this description. Zeng and co-workers have been pioneers in this area,^98^ developing transport system **16** (Figure 11), a so-called “molecular ion fisher.” The cholesterol unit was used to anchor the system, while the flexible linker to the benzo-18-crown-6 K^+^ binding motif facilitated transport by this unconventional mechanism.^99^ Transporters with different anchoring architectures have been reported, based on a single cholesterol lock,^99^ as well as systems with a membrane-spanning cross-beam containing two cholesterol units **17**.^100,102^ A computational study found that H-bonding between the cholesterol and the phosphate of a lipid restricted lateral translocation, rationalizing the efficiency of this anchor group.^103^ Interestingly, after prolonged investigations, no single-channel current traces were obtained for the moncholesterol **16** or a bis-cholesterol anchored system in BLM conductance experiments, indicating that ions were not transported through well-defined channels.^99,102^ Ill-defined and transient single-channel current traces were observed for the molecular swing transporter **17** in BLM experiments.^100^ This highlights that there is a broad spectrum of possible mechanisms of ion transport, varying from the ill-defined pathway of mobile carriers to the well-defined path of ion translocation through an ion channel with the unimolecular anchored carrier mechanism positioned somewhere between the two.

Exchanging the substituent on a tetrasubstituted anchored carrier provided insight into the effect of increasing the number of carrier arms.^101^ Derivative **18a** with three indolyl anchoring groups and one benzo-18-crown-6 receptor displayed so-called “molecular fishing” activity, while the anchored carrier **18b** (two anchors, two receptor) displayed >30 times greater activity. The carrier arms therefore operated cooperatively, but whether both receptors bound the ion simultaneously to transport via a molecular fishing mechanism, or whether an exchange process occurred within the membrane to transport via a relay mechanism was difficult to confirm. Since no single channel behavior was observed in BLM experiments, transport activity was not due to a channel mechanism and thus can be attributed to an anchored carrier mechanism.

In a similar approach, the same group reported an octa-substituted C_60_ buckyball with crown ether receptors which operated via an anchored carrier mechanism.^106^ The selectivity for the cation transported could be tuned based on the size of the crown ether macrocycle. In these anchored carrier systems, the flexible linker between the receptor and anchoring group enabled transport by allowing mobility of the pendant receptor. In contrast, crown ethers appended directly to a membrane-spanning anchor with short rigid linkers have previously been shown to follow a strict channel-type mechanism of transport.^107^

**Figure 12:**
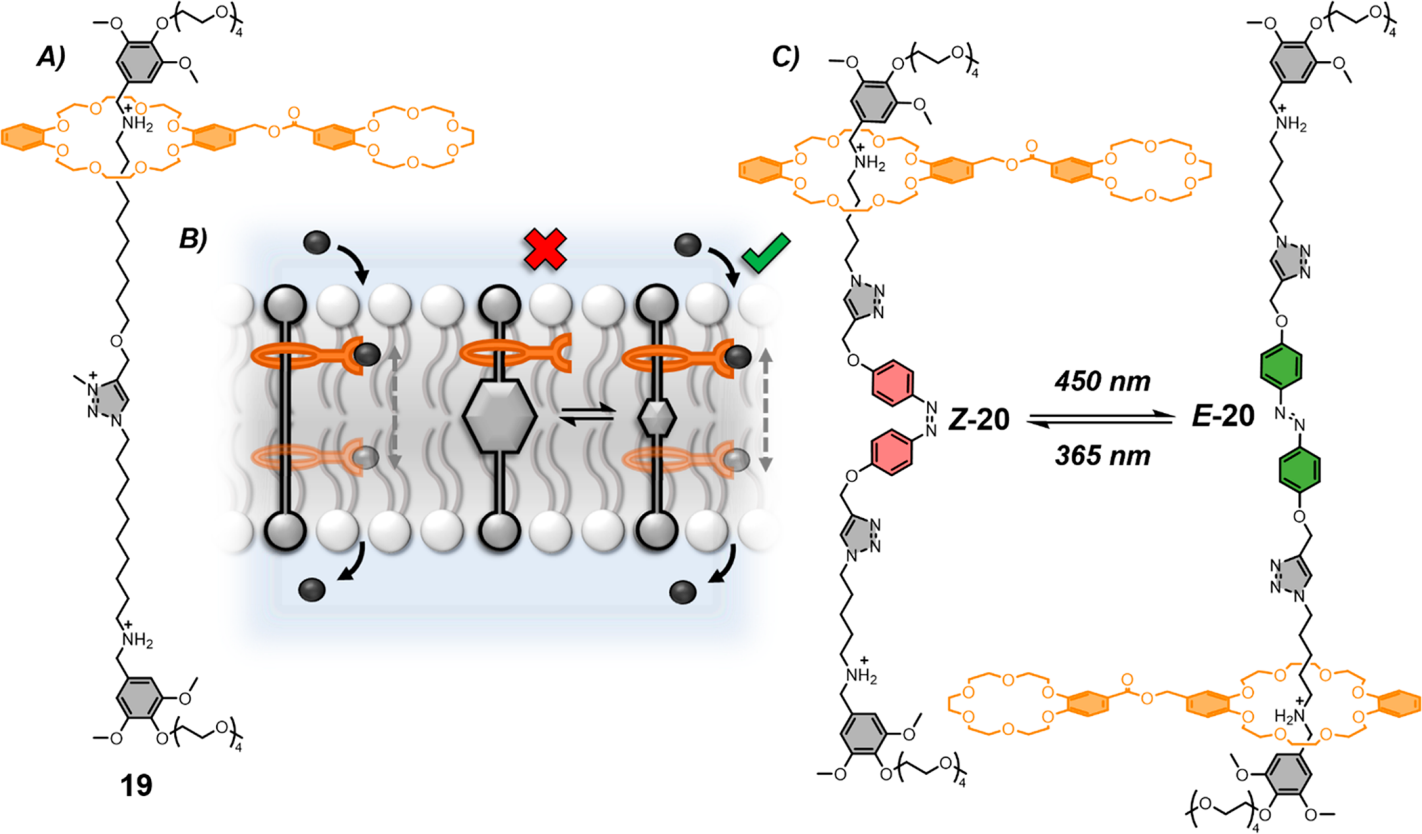
Unimolecular anchored carriers based on a [2]rotaxane architecture. A. Structure of [2]rotaxane cation transporter **19**.^104^ B. Schematic of [2]rotaxane ion transporters where shuttling motion facilitates ion transport. C. Azobenzene isomerization in the axle of [2]rotaxane **20** allows photoswitching of transport activity between the off state (***Z*****-20**) and on state (***E*****-20**).^105^

Employing a mechanical bond to link the carrier unit to the anchoring group was demonstrated by Bao, Zhu, and co-workers in 2018, who reported a [2]rotaxane shuttle **19** able to transport K^+^ via a shuttle-type mechanism (Figure 12).^104^ The activity of **19** was found to be linearly dependent on the concentration of the transporter in the bilayer, indicative of a single molecule being responsible for transport. Regular square-like signals with long opening times and ohmic I–V profiles in BLM experiments suggested a stable channel may form within the bilayer. However, a unimolecular dependence of transport activity and the reduced transport activity with decreasing shuttling rate implied that the mobility of the carrier arm along the axle of the rotaxane was responsible for the transport activity.^108^ Subsequent interlocked unimolecular anchored carriers have been reported, which have unique axle structures and an amide-linked crown ether macrocycle carrier arm.^109,110^ Interestingly, the rigid *para*-tetraphenylene axle derivative (EC_50_ = 1.75 mol %)^109^ displayed enhanced activity relative to the flexible [2]rotaxane **19** (EC_50_ = 3 mol %).^104^ Increasing the number of carrier arms in the interlocked transporter was shown to improve the transport activity, with EC_50_ values of 4.3 mol % and 2.0 mol % for the [2]rotaxane and [3]rotaxane, respectively.^110^ As with the unimolecular anchored carrier system **18b**, determination of whether both receptors bind the ion simultaneously to transport via a molecular fishing mechanism, or whether an exchange process occurred within the membrane to transport via a relay mechanism, was not confirmed.

A later related system combined an interlocked architecture with a molecular switch to facilitate photogated shuttling motion for the regulation of transport activity.^105^ An azobenzene was incorporated within the membrane-spanning axle, such that shuttling of the macrocycle, and hence mobility of the anchored carrier arm, could be restricted through photoswitching the azobenzene moiety to ***Z*****-20** to diminish cation transport rates. The system was shown to be fully reversible, with in situ light-gated transmembrane activity measured in LUVs and BLM experiments. Single-channel conductance experiments showed regular square-like signals after irradiation with 450 nm visible light forming ***E*****-20**, while the ***Z*****-20** displayed no current signal after long detection times. This unique molecular-machine-inspired anchored carrier transporter combines the properties of molecular switches with the dynamic motion of interlocked components to control transport across lipid membranes. The structure of the axle is key to its function as an anchor, orienting the molecule as a bolaamphiphile to span the membrane, and enabling the shuttling motion of the [2]rotaxane to transport ions across the membrane

### Relay Transporters

**Figure 13:**
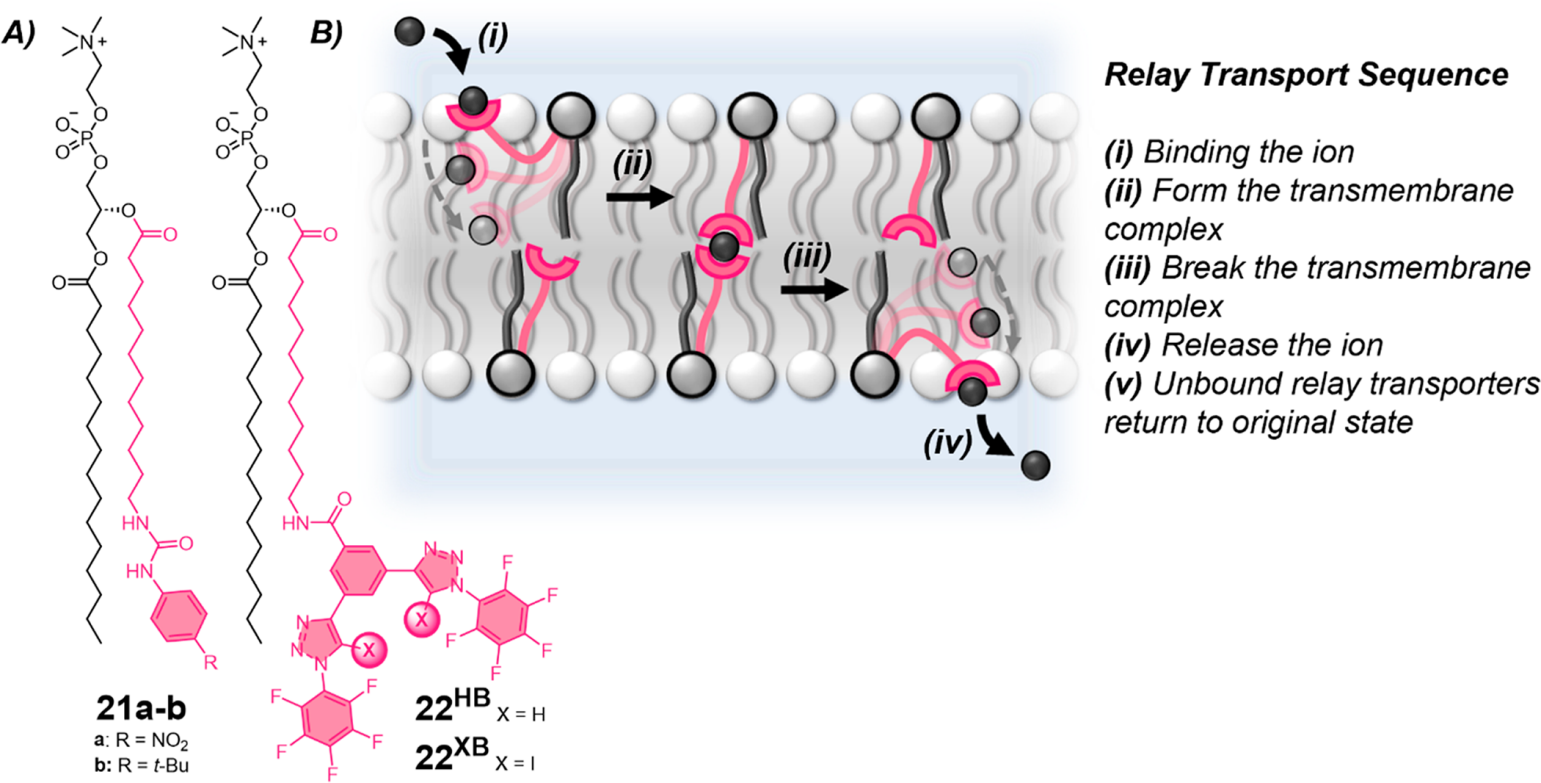
Relay transporters. A. Smith’s urea relay transporter **21**,^111^ Langton’s bis-iodotriazole hydrogen (**22**^**HB**^), and halogen bonding (**22**^**XB**^) relay transporters.^112^ B. Schematic of the relay transport mechanism.

Relay transporters operate via the exchange of ions between transporters located in opposite leaflets of a lipid bilayer membrane. The relay transport mechanism was first reported by Smith and co-workers in 2008, who demonstrated that a urea-appended phospholipid derivative **21** facilitated ion transport by passing anions between transporters immobilized in opposite leaflets of the bilayer (Figure 13).^111^ They established the key principles of this unique transport mechanism, demonstrating that relay transporters must be present in both leaflets of the bilayer with no activity observed when loaded into only one leaflet of the membrane. The electron-withdrawing nitro substituent of **21a** significantly improved transport activity relative to the *t*-butyl group (**21b**), as a result of the increased binding strength for anions. An extensive survey of different phospholipids of varying alkyl chain lengths was also conducted to explore the effect of bilayer hydrophobic thicknesses on transport rates. Activity was found to incrementally decrease with increasing phospholipid alkyl chain length from 14 to 18 carbons and was completely suppressed for lipid alkyl chain lengths of 20 to 24 carbons. Notably, this trend contrasts with that typically observed for ion channels which display a bell-shaped relationship, where optimal activity is observed for channel structures that matched the thickness of the bilayer,^113^ and unlike that of mobile carriers, which is either invariant with membrane thickness when ion binding is rate-limiting,^55^ or linearly decreased with increasing hydrophobic thickness when translocation of the carrier–ion complex is rate-limiting.^114,115^

Langton and co-workers subsequently reported a relay transporter with a bis-iodotriazole halogen bonding (XB) anion receptor.^112^ The relay transporter **22**^**XB**^ showed enhanced transport activity with an EC_50_ value of 0.18 mol % with respect to lipid in POPC LUVs, compared to ∼ 2 mol % in POPC/cholesterol (7:3) for Smith’s urea derivative **21a**.^111^ In the presence of the protonophore FCCP, the activity of the halogen-bonding relay transporter **22**^**XB**^ was significantly enhanced (EC_50_ = 0.036 mol %), as a result of appreciable Cl^–^ > H^+^/OH^–^ selectivity. The prototriazole derivative **22**^**HB**^ exhibited efficient transport activity (EC_50_ = 0.18 mol %, in POPC LUVs) but no transport enhancement with FCCP, due to the hydrogen bond donors showing no Cl^–^ > H^+^/OH^–^ selectivity. Phospholipid-based ion transporters have the added benefit of preferential solubility in physiological environments to improve the likelihood of pharmaceutical success. Relay **22**^**XB**^ demonstrates that otherwise highly hydrophobic halogen bonding anion receptors can be incorporated into amphiphilic ion transporters, while maintaining the intrinsic Cl^–^ > OH^–^ selectivity which is vital for the application of synthetic transporters as therapeutics.

**Figure 14:**
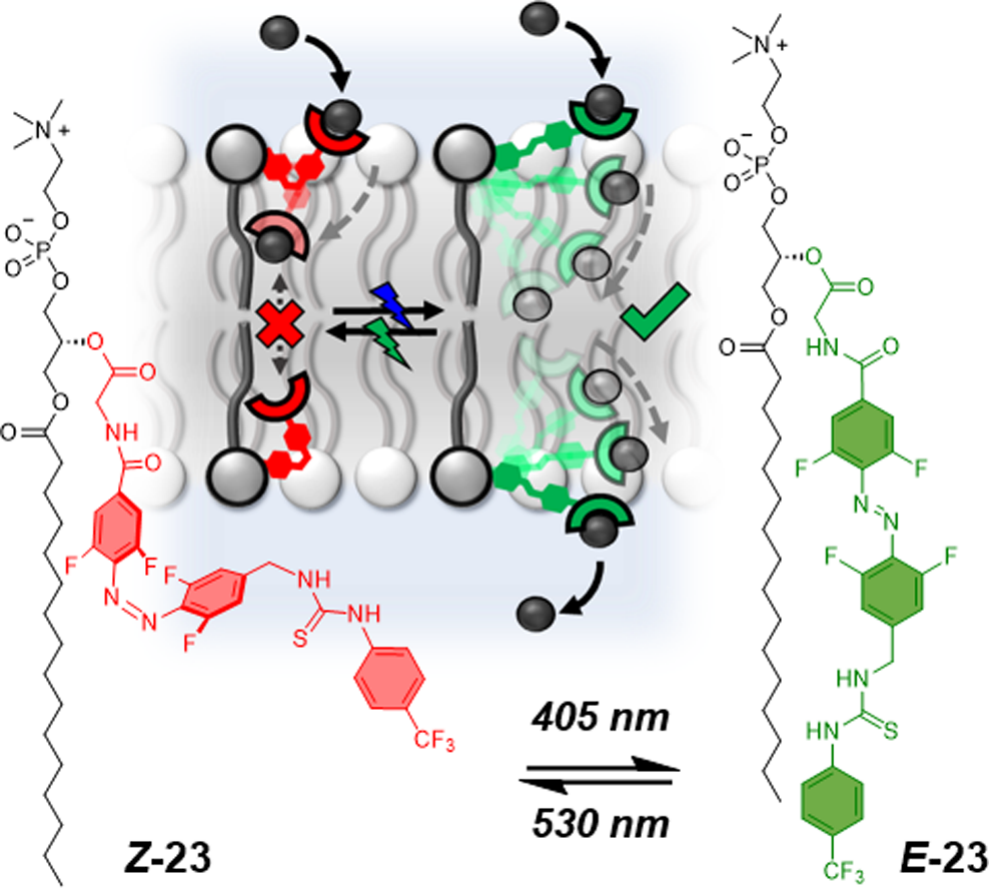
Photoswitchable relay transporter. Schematic representation of photoregulated relay transport activity, and Langton’s tetra-fluoroazobenzene thiourea relay transporter **23**.^116^

The same group also demonstrated that the incorporation of a molecular switch into the carrier arm of a relay transporter could be employed to reversibly control the length of the relay arm, in turn regulating the transport activity (Figure 14).^116^ A visible light photoswitchable tetra-fluoroazobenzene^117^ was incorporated into a phospholipid-based relay transporter, which could be efficiently switched between the *E*- and *Z*-isomers using green and blue light within the membrane, with the same photostationary state distribution as was observed in solution. This system demonstrated the critical role of the “telescopic” arm length for controlling the relay transport process: the gap between the thiourea anion-binding sites of transporters on opposite sides of the membrane must be sufficiently small to mediate transport for ***E*****-23**, but also sufficiently large for ***Z*****-23** to suppress transport in the off state. Notably, this mechanism requires the unprecedented control of multiple molecular machine-like components positioned on opposite sides of a membrane, which work together in a cooperative manner to facilitate transport.

## CONTROLLED RELEASE OF CARGO FROM LIPID BOUND COMPARTMENTS

In the previous sections, we have described how molecular machine components and nanomechanical motions of anchored carriers have been used to control transport of cargo across lipid bilayer membranes through selective binding interactions. Molecular machines have also been shown to control the disruption of membranes to facilitate the nonspecific release of cargo from lipid-bound compartments.^118^ Indeed, extensive research has been conducted into the development of compounds which disrupt cellular membranes for antiviral and antimicrobial activity,^119,120^ as well as for targeted drug delivery.^121−125^ This perspective will focus on the molecular switches and motors that exploit controlled nanomechanical motion to disrupt both cellular and model membranes.

### Membrane Disruption with Molecular Switches

**Figure 15:**
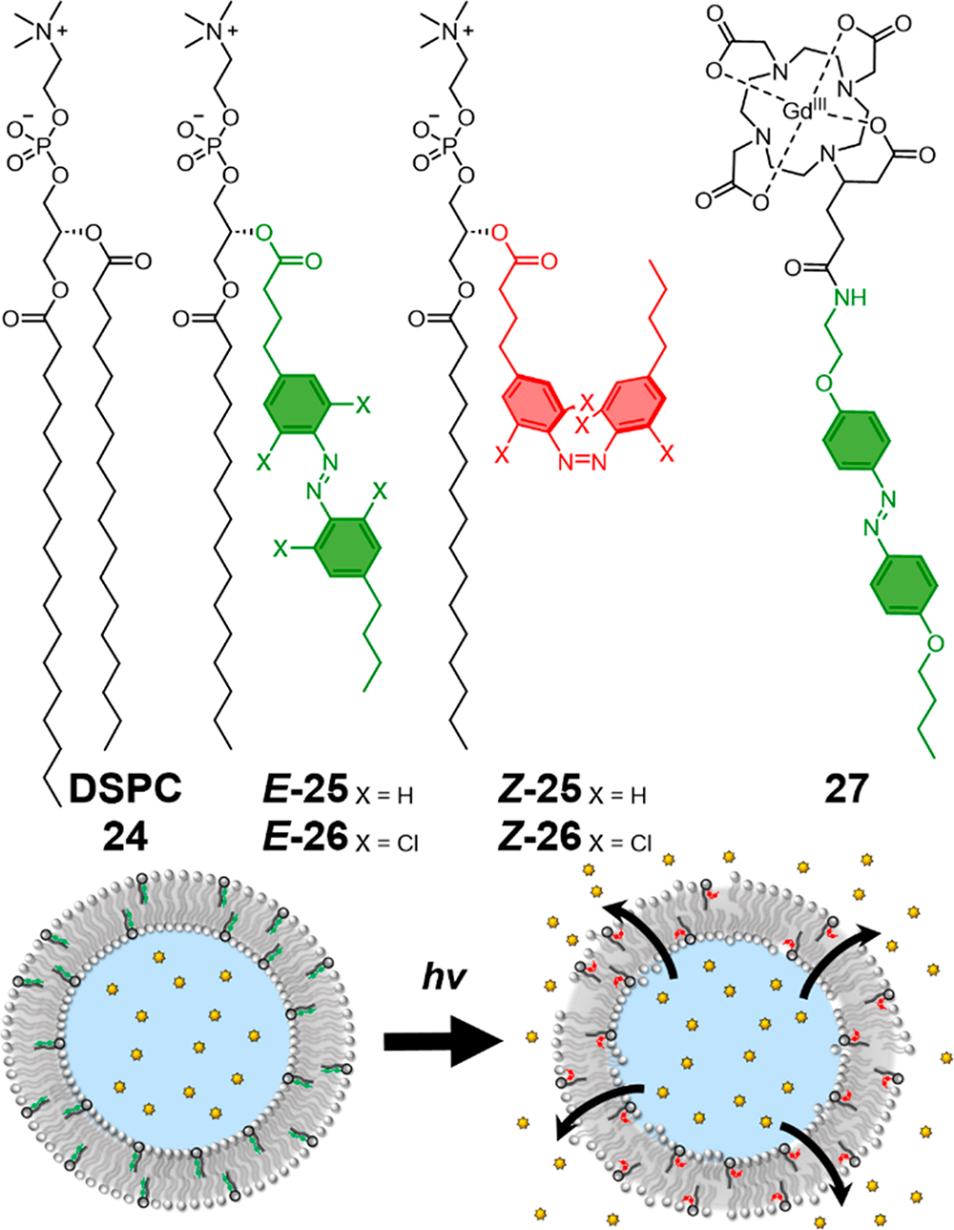
Photoswitches for lipid membrane disruption. Top: structure of DSPC lipid **24**, UV-responsive azoPC **25**,^125^ red-light-responsive red-azoPC **26**,^130^ and ionizing-radiation-responsive gadolinium complex **27**.^133^ Bottom: schematic of phototriggered release of cargo from a lipid compartment.

The field of photolipids is well established^126,127^ for the light-controlled release of cargo from the lumen^128^ or the membrane of vesicles.^122^ In general, molecular switches in the linear *E*-configuration (***E*****-25**) align well with other lipids in the bilayer, while photoswitching to the bent *Z*-configuration (***Z*****-25**) disrupts lipid packing and causes the release of the cargo (Figure 15).^129^ In recent years, to improve the biocompatibility of these vesicle drug delivery systems (which are typically activated with UV light with poor biocompatibility and tissue penetration), there has been a drive to red-shift the photoswitching process for operation within the therapeutic window.

Trauner and co-workers introduced the tetra-chloroazobenzene phospholipid **26** which has been shown to mediate the photocontrolled release of doxorubicin from distearoylphosphatidylcholine (DSPC)/cholesterol vesicles with 630 nm/465 nm light in vivo in zebrafish embryos.^125,130^ Encapsulating lanthanide upconversion nanoparticles within the lumen of vesicles is another method that has been shown to enable long-wavelength photoswitching of azobenzene photolipids.^131,132^ These nanoparticles absorb 980 nm near-infrared photons and re-emit shorter wavelengths of light, including UV (∼360 nm) and blue (∼450 nm), which was shown to isomerize the azobenzene, releasing doxorubicin. A gadolinium–azobenzene conjugate **27** has recently been reported, which can be switched with ionizing radiation.^133^ The lanthanide initially absorbs high-energy photons and mediated switching through energy transfer to the azobenzene core. This radio-switch amphiphile demonstrated ionizing radiation could induce molecular rearrangement of the azobenzene to disrupt cellular membranes and trigger a cytotoxic effect.

**Figure 16:**
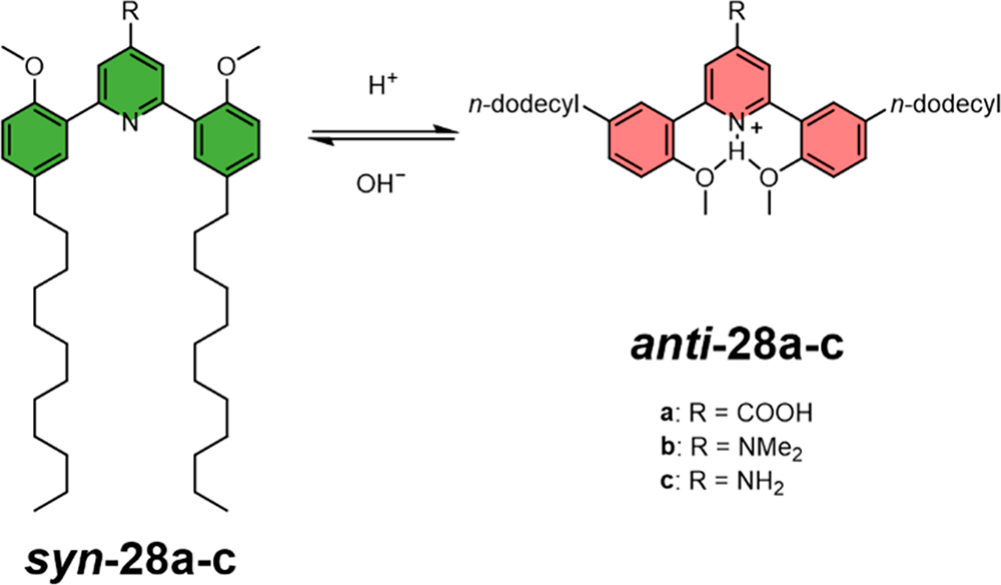
pH-Responsive bis(methoxyphenyl)-pyridine switch for triggered membranes disruptions. The various R groups of **28a–c** control the pH at which conformational switching from ***syn***-**28** to ***anti***-**28** occurs.^135^

Molecular switches responsive to pH have also been demonstrated to control the release of cargo from vesicles.^134^ The Leblond group have developed a bis(methoxyphenyl)-pyridine-based pH-responsive synthetic lipid **28** which displayed large amplitude conformational switching (Figure 16).^135^ In the neutral state, the molecule adopted a closed conformation where the long alkyl chains of ***syn*-28** were in a parallel arrangement, which allowed efficient incorporation and packing within a DSPC membrane. Protonation of the pyridine nitrogen at low pH induced molecular switching to the open ***anti*-28** conformation, which significantly disrupted the bilayer of LUVs and triggered the release of encapsulated cargo. The pH-triggered release of sulforhodamine B from **28b**-loaded DSPC LUVs was shown to be actuated by the acidic interior of HeLa cells, which affects the rapid endosomal release of cargo into live cells. A subsequent report demonstrated that the same strategy could be used for the delivery of small interfering RNA (siRNA) into HeLa cells.^136^ This technology has promise for treating a range of diseases, due to the ability of siRNA to regulate gene expression.^137^

### Membrane Release Triggered by Molecular Motors

Amphiphilic molecular motors have been shown to reversibly reorganize between different self-assembled structures in aqueous media, controlled by the isomerization of the motor.^138−140^ However, only a few examples have been interfaced with membranes to perform a function.^141^ Tour and co-workers first demonstrated that membrane-anchored molecular motors could disrupt cellular membranes for the targeted killing of cancer cells.^142^ Their results suggested that the unidirectional rotation of the molecular motors could be exploited to form transient pores in both model and cellular membranes. Under 355 nm irradiation, **29a** increased the permeability of cellular membranes, leading to necrosis of human prostate adenocarcinoma cell (PC-3). The demethylated derivative **29b**, incapable of unidirectional rotation, displayed a reduced cytotoxic effect. However, a recent study from Pohl, Antonenko, and co-workers suggested that the mechanism of action of motor **29a** is the generation of singlet oxygen by the photoexcited state of the motor, which caused oxidative damage of unsaturated lipids and destabilized the membrane.^143^ This was explored by conducting experiments in giant unilamellar vesicles—those prepared with dipalmitoylphosphatidylcholine (DOPC, an unsaturated lipid) were susceptible to motor-induced membrane disruption after irradiation; however, those prepared with 1,2-diphytanyl-*sn*-glycero-3-phosphocholine (DPhPC, a saturated lipid) were inert to molecular motor action.

**Figure 17:**
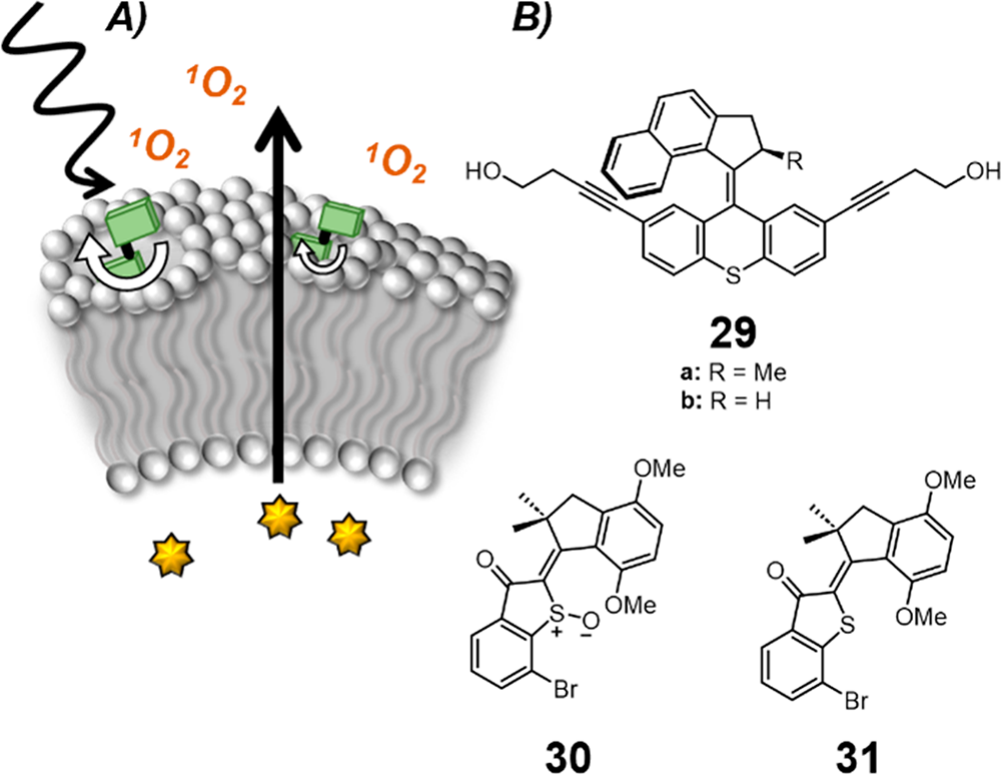
Molecular motors used to disrupt cellular membranes. A. Schematic of motor-induced membrane rupture through generation of ROS or via the proposed nanomechanical motion drilling holes in membranes. B. Tour’s fast rotating motor (**29a**), switch (**29b**).^142^ The hemithioindigo motor (**30**) and switch (**31**) disrupt bacterial membranes through oxidative damage.^144^

Tour and co-workers have also explored hemithioindigo-based visible-light-activated molecular motors for their antibacterial activity (Figure 17).^144^ The sulfoxide hemithioindigo motor **30** displayed some antibacterial activity upon 455 nm irradiation, which promoted 360° unidirectional rotation. However, the related sulfide hemithioindigo switch **31** that is incapable of complete rotation was found to be more potent, again pointing to the generation of reactive oxygen species (ROS) playing a role in the mechanism of action of these biologically active molecular machines.

Feringa and co-workers have investigated the use of molecular motors for the controlled release of cargo from lipid-bound compartments, with the view to develop targeted drug delivery vehicles.^145^ Incorporating motors within amphiphilic molecules has been shown to generate micelle and vesicle self-assembled structures, to allow the investigation of their dynamic behavior in biologically relevant media.^146,147^ Hydrophobic motors have also been incorporated within lipid membranes, where 365 nm light irradiation led to the release of molecular cargo from DOPC (unsaturated lipid) vesicles.^148^ Tour and co-workers have also demonstrated that molecular motors can be used in combination with therapeutic agents to improve their cellular internalization.^149^ The action of **29a** increased the sensitivity of *Klebsiella pneumoniae* to Meropenem, thereby improving the biological effect of the antibiotic. While the mechanism of action of cellular targeting molecular motors may not have been fully elucidated,^150^ their apparent efficacy within a biological environment is certainly promising and worthy of detailed investigation, and provides inspiration for the development of further biocompatible molecular rotors in the future.

## STRATEGIES AND CHALLENGES FOR MEMBRANE TARGETING AND TOWARD APPLICATIONS IN CELLS

In cellular biology, membrane labeling for imaging is typically achieved using lipophilic fluorophores with pendant aliphatic chains, which spontaneously intercalate into the lipid bilayer.^151^ Uptake of hydrophobic molecular machines into artificial membranes remains the main method to ensure membrane confinement of these systems and is often achieved with bioinspired anchoring groups, such as cholesterol, farnesyl, or functionalized lipids themselves. Alternatively, in many cases a sufficiently hydrophobic core scaffold of a molecular machine derived from entirely artificial structures—a Feringa type rotor for instance—is more than sufficient for facilitating membrane uptake.^51^ Influencing the orientation and position of a molecular machine within the lipid bilayer, for example, where it sits in relation to the interface, has been typically achieved by careful control over the positioning of hydrophilic and hydrophobic motifs within the system. For instance, Kinbara’s mechanosensitive multipass channel (Figure 5D) uses a combination of a hydrophobic core to embed within the membrane interior, and polyethylene glycol chains which reside at the membrane interface, in order to control the required membrane-spanning orientation.^42^ Similarly, Zeng has made use of hydroxylated cholesterol motifs to preferentially locate a molecular ion fisher at the membrane interface (Figure 11).^99^ Controlling the orientation of molecular transporter machines in membranes has typically been achieved through designing amphiphilic systems, such that when added to the membranes of preformed vesicles, directional insertion of the hydrophobic portion of the machine occurs preferentially. This is demonstrated in Smith’s and Langton’s relay transporter systems (Figure 13 and 14), in which the amphiphilic lipid anchor ensures directional uptake into the membrane.^111,112,116^

More generally, there are a range of challenges to be overcome to interface molecular machines with living cells. Indeed, some of the recent progress in the field of ion transport systems has arguably been motivated by the potential longer-term translation of these synthetic molecules as therapeutics, yet the application of synthetic ion transporters has rarely progressed beyond model systems within the lab. Further work is required to understand how results in model vesicle or BLM membranes translate into comparable behavior in cells, animal models, and in vivo. Deliverability is likely to be a key challenge to address, and cell membrane uptake of ionophores in general is currently underexplored. Indeed, a study by Sheppard and co-workers investigated a wide range of potent hydrogen-bonding anionophores in vesicles and Fischer rat thyroid (FRT) cells,^152^ and found that many of the synthetic transporters had poor deliverability to the membrane. They also demonstrated that cellular uptake could be enhanced by adding transporters in the presence of lipid to form water-soluble aggregates. Liposome-based delivery systems have also shown some promise in enhancing uptake into giant unilamellar vesicles,^153^ and this approach is certainly worthy of investigation for the delivery of molecular machines to cells. In general, a thorough investigation into whether the current strategies for facilitating uptake into model membranes, as discussed above, translate to cells is now required. Such studies will also need to explore the time residence of such machines within the bilayer, and methods to slow down endocytic elimination.^154^

To date there are few studies in which molecular machine ion transporters, including switchable transporters,^88,97^ interlocked carriers,^62^ or molecular rotors^52,142^ have been studied in cellular systems, and thus there is little current understanding of how such systems may be usefully employed in this context. Jeong and co-workers investigated photoswitchable anion mobile carriers **12** (Figure 9) in FRT cells.^88^ These investigations revealed that the *para*-substituted transporters **12f** and **12g** displayed no activity as either the *E*- or *Z*-isomers (unlike in POPC vesicles), while in contrast, the analogous *meta*-substituted derivatives displayed transport activity in the compact *Z*-isomer and not in the extended *E*-isomer in FRT cells, in line with observation in LUVs. The lack of activity for the *para*-substituted transporters was speculated to be due to protein binding inhibiting activity but could also plausibly arise from low deliverability to the cellular membrane and highlights the challenges in translating experiments in model systems through to cells. The disruption of cellular homeostasis through perturbation of ion concentration gradients by synthetic transporters has been shown to induce cell death,^155^ and the measurement of cell viability has been used to provide an indirect measure of transport activity. Such experiments have been conducted with the anchored carrier **17** and a benzo-18-crown-6 analogue in human primary glioblastoma cancer cells.^100^ The efficacy of the anchored carrier transport system in inducing cell death suggests that the abiotic anchored carrier transport mechanism can operate in cellular membranes. This is the only example to date of an anchored carrier having been tested in cellular membranes but showcases the potential of abiotic mechanisms of molecular machine-like transporters for facilitating transport in cellular systems.

## CONCLUSIONS AND OUTLOOK

Nature confines its protein molecular machinery within lipid bilayer membranes to orient and exploit compartmentalization and transmembrane ion gradients. Numerous different approaches to incorporating molecular machines, in the broadest sense, within lipid bilayers to control ion transport have been developed. Many of these are derived from molecular machines first explored in the solution phase, such as switches, rotors, and rotaxanes. The lipid bilayer environment provides a means of confining molecular machines in an organized manner, with a high local concentration promoting intermolecular interactions and hence the potential for cooperative function. As such, molecules which in bulk solution may not display any function, such as relay transporters, can be immobilized within the bilayer and work cooperatively to control transmembrane transport. The biophysical properties of lipid bilayer membranes, such as membrane tension—a property that is evidently absent in the solution phase—have been demonstrated to regulate the operation of membrane-bound molecular machines and provide a further level of control to these systems. More generally, embedding molecular machines within the hydrophobic interior of the bilayer provides a useful method for interfacing organic molecules with aqueous, biologically relevant environments. Indeed, employing molecular machines for biomedical applications already appears to be a fruitful area of research, with fundamental studies in living cells demonstrating the benefit of their abiotic mechanisms of action.

Beyond controlling transmembrane transport, molecular machines in lipid bilayers may also serve to mediate other functions, including signal transduction,^156−159^ catalysis,^160−162^ and sensing applications.^163,164^ Advancements in these related fields are likely to serve as inspiration for the development of molecular-machine-based transmembrane transporters. Indeed, the control of membrane incorporation, orientation, flip-flop, and residence time are common challenges within all of these areas of study. For example, Matile’s membrane tension responsive fluorescent flipper probes, which change conformation and hence fluorescent output within the membrane,^165−167^ provide strategies to internalization and targeting of specific cellular membranes which could in future be adapted to molecular transport machines. How best to interface synthetic molecular machines with membranes, with control over membrane targeting, orientation, and retention, is one of the most key current challenges to be addressed for the translation of molecular machines into biological environments.

The future directions for this field of research will surely also include the development of out-of-equilibrium function, active transport, and chemically fuelled membrane-bound molecular machines. The complexity of biological membrane-bound machines, such as ATP synthase, will continue to serve as a benchmark for inspiring chemists to develop synthetic systems of increased complexity and sophistication. So far, no synthetic systems have been developed in which nanomechanical motion is driven by a concentration gradient across the membrane. Nor have any molecular machines been reported which facilitate active transport of an analyte against its concentration gradient, driven by a chemical fuel,^168,169^ in a manner reminiscent of the mode of action of ATP-ases. Indeed, active transport across lipid membranes using synthetic transporters in general remains extremely challenging, with only two examples using a synthetic photoredox carrier^170^ or channel^48^ system reported. Finally, interfacing synthetic molecular machines with biological components to access novel biohybrid systems^171^ is likely to be the focus of research to come, and is set to yield exciting opportunities for new biomedical applications.

## References

[ref1] Sauvage J.-P. From Chemical Topology to Molecular Machines (Nobel Lecture). Angew. Chem., Int. Ed. 2017, 56 (37), 11080–11093. 10.1002/anie.201702992. 28632333

[ref2] Stoddart J. F. Mechanically Interlocked Molecules (MIMs)-Molecular Shuttles, Switches, and Machines (Nobel Lecture). Angew. Chem., Int. Ed. 2017, 56 (37), 11094–11125. 10.1002/anie.201703216. 28815900

[ref3] Feringa B. L. The Art of Building Small: From Molecular Switches to Motors (Nobel Lecture). Angew. Chem., Int. Ed. 2017, 56 (37), 11060–11078. 10.1002/anie.201702979. 28851050

[ref4] Gadsby D. C. Ion Channels versus Ion Pumps: The Principal Difference, in Principle. Nat. Rev. Mol. Cell Biol. 2009, 10 (5), 344–352. 10.1038/nrm2668. 19339978 PMC2742554

[ref5] Aprahamian I. The Future of Molecular Machines. ACS Cent. Sci. 2020, 6 (3), 347–358. 10.1021/acscentsci.0c00064. 32232135 PMC7099591

[ref6] Corra S.; Curcio M.; Baroncini M.; Silvi S.; Credi A. Photoactivated Artificial Molecular Machines That Can Perform Tasks. Adv. Mater. 2020, 32 (20), 1906064 10.1002/adma.201906064. 31957172

[ref7] Browne W. R.; Feringa B. L. Making Molecular Machines Work. Nat. Nanotech. 2006, 1 (1), 25–35. 10.1038/nnano.2006.45. 18654138

[ref8] Erbas-Cakmak S.; Leigh D. A.; McTernan C. T.; Nussbaumer A. L. Artificial Molecular Machines. Chem. Rev. 2015, 115 (18), 10081–10206. 10.1021/acs.chemrev.5b00146. 26346838 PMC4585175

[ref9] Watson M. A.; Cockroft S. L. Man-Made Molecular Machines: Membrane Bound. Chem. Soc. Rev. 2016, 45 (22), 6118–6129. 10.1039/C5CS00874C. 26932423

[ref10] Davis J. T.; Gale P. A.; Quesada R. Advances in Anion Transport and Supramolecular Medicinal Chemistry. Chem. Soc. Rev. 2020, 49 (16), 6056–6086. 10.1039/C9CS00662A. 32692794

[ref11] Bickerton L. E.; Johnson T. G.; Kerckhoffs A.; Langton M. J. Supramolecular Chemistry in Lipid Bilayer Membranes. Chem. Sci. 2021, 12, 11252–11274. 10.1039/D1SC03545B. 34567493 PMC8409493

[ref12] Ahmad M.; Gartland S. A.; Langton M. J. Photo- and Redox-Regulated Transmembrane Ion Transporters. Angew. Chem., Int. Ed. 2023, 62, e202308842 10.1002/anie.202308842. 37478126

[ref13] de Jong J.; Bos J. E.; Wezenberg S. J. Stimulus-Controlled Anion Binding and Transport by Synthetic Receptors. Chem. Rev. 2023, 123 (13), 8530–8574. 10.1021/acs.chemrev.3c00039. 37342028 PMC10347431

[ref14] Langton M. J. Engineering of Stimuli-Responsive Lipid-Bilayer Membranes Using Supramolecular Systems. Nat. Rev. Chem. 2021, 5 (1), 46–61. 10.1038/s41570-020-00233-6. 37118103

[ref15] Yang K.; Kotak H. A.; Haynes C. J. E. Metal-Organic Ion Transport Systems. Coord. Chem. Rev. 2022, 470, 214705 10.1016/j.ccr.2022.214705.

[ref16] Zheng S. P.; Huang L. B.; Sun Z.; Barboiu M. Self-Assembled Artificial Ion-Channels toward Natural Selection of Functions. Angew. Chem., Int. Ed. 2021, 60 (2), 566–597. 10.1002/anie.201915287. 32212308

[ref17] Barba-Bon A.; Nilam M.; Hennig A. Supramolecular Chemistry in the Biomembrane. ChemBioChem 2020, 21 (7), 886–910. 10.1002/cbic.201900646. 31803982 PMC7187467

[ref18] Davis A. P.; Sheppard D. N.; Smith B. D. Development of Synthetic Membrane Transporters for Anions. Chem. Soc. Rev. 2007, 36 (2), 348–357. 10.1039/B512651G. 17264935 PMC2854546

[ref19] Docker A.; Langton M. J. Transmembrane Anion Transport Mediated by Sigma-Hole Interactions. Trends Chem 2023, 5, 792–794. 10.1016/j.trechm.2023.06.001.

[ref20] Murphy B. L.; Gabbaï F. P. Binding, Sensing, And Transporting Anions with Pnictogen Bonds: The Case of Organoantimony Lewis Acids. J. Am. Chem. Soc. 2023, 145 (36), 19458–19477. 10.1021/jacs.3c06991. 37647531 PMC10863067

[ref21] Jowett L. A.; Gale P. A. Supramolecular Methods: The Chloride/Nitrate Transmembrane Exchange Assay. Supramol. Chem. 2019, 31 (5), 297–312. 10.1080/10610278.2019.1574017.

[ref22] Gilchrist A. M.; Wang P.; Carreira-Barral I.; Alonso-Carrillo D.; Wu X.; Quesada R.; Gale P. A. Supramolecular Methods: The 8-Hydroxypyrene-1,3,6-Trisulfonic Acid (HPTS) Transport Assay. Supramol. Chem. 2021, 33 (7), 325–344. 10.1080/10610278.2021.1999956.

[ref23] McNally B. A.; Koulov A. V.; Smith B. D.; Joos J. B.; Davis A. P. A Fluorescent Assay for Chloride Transport; Identification of a Synthetic Anionophore with Improved Activity. Chem. Commun. 2005, 1087–1089. 10.1039/b414589e. 15719125

[ref24] Chvojka M.; Singh A.; Cataldo A.; Torres-Huerta A.; Konopka M.; Šindelář V.; Valkenier H. The Lucigenin Assay: Measuring Anion Transport in Lipid Vesicles. Analysis & Sensing 2023, e202300044 10.1002/anse.202300044.

[ref25] Gokel G. W. Hydraphiles: Design, Synthesis and Analysis of a Familyof Synthetic, Cation-Conducting Channels. Chem. Commun. 2000, 1–9. 10.1039/a903825f.

[ref26] Gokel G. W.; Negin S. Synthetic Ion Channels: From Pores to Biological Applications. Acc. Chem. Res. 2013, 46 (12), 2824–2833. 10.1021/ar400026x. 23738778

[ref27] Baroncini M.; Silvi S.; Credi A. Photo- And Redox-Driven Artificial Molecular Motors. Chem. Rev. 2020, 120 (1), 200–268. 10.1021/acs.chemrev.9b00291. 31415169

[ref28] Fehrentz T.; Schönberger M.; Trauner D. Optochemical Genetics. Angew. Chem., Int. Ed. 2011, 50 (51), 12156–12182. 10.1002/anie.201103236. 22109984

[ref29] Volarić J.; Szymanski W.; Simeth N. A.; Feringa B. L. Molecular Photoswitches in Aqueous Environments. Chem. Soc. Rev. 2021, 50 (22), 12377–12449. 10.1039/D0CS00547A. 34590636 PMC8591629

[ref30] Mukhopadhyay T. K.; Morstein J.; Trauner D. Photopharmacological Control of Cell Signaling with Photoswitchable Lipids. Curr. Opin. Pharmacol. 2022, 63, 102202 10.1016/j.coph.2022.102202. 35278838

[ref31] August D. P.; Borsley S.; Cockroft S. L.; della Sala F.; Leigh D. A.; Webb S. J. Transmembrane Ion Channels Formed by a Star of David [2]Catenane and a Molecular Pentafoil Knot. J. Am. Chem. Soc. 2020, 142 (44), 18859–18865. 10.1021/jacs.0c07977. 33084320 PMC7745878

[ref32] Haynes C. J. E.; Zhu J.; Chimerel C.; Hernández-Ainsa S.; Riddell I. A.; Ronson T. K.; Keyser U. F.; Nitschke J. R. Blockable Zn10L15 Ion Channels through Subcomponent Self-Assembly. Angew. Chem., Int. Ed. 2017, 56 (48), 15388–15392. 10.1002/anie.201709544. 29024266

[ref33] Xiao Q.; Haoyang W. W.; Lin T.; Li Z. T.; Zhang D. W.; Hou J. L. Unimolecular Artificial Transmembrane Channels Showing Reversible Ligand-Gating Behavior. Chem. Commun. 2021, 57 (7), 863–866. 10.1039/D0CC06974D. 33439165

[ref34] Yan T.; Liu S.; Li C.; Xu J.; Yu S.; Wang T.; Sun H.; Liu J. Flexible Single-Chain-Heteropolymer-Derived Transmembrane Ion Channels with High K+ Selectivity and Tunable PH-Gated Characteristics. Angew. Chem., Int. Ed. 2022, 61 (42), e202210214 10.1002/anie.202210214. 36039469

[ref35] Mamad-Hemouch H.; Ramoul H.; Abou Taha M.; Bacri L.; Huin C.; Przybylski C.; Oukhaled A.; Thiébot B.; Patriarche G.; Jarroux N.; Pelta J. Biomimetic Nanotubes Based on Cyclodextrins for Ion-Channel Applications. Nano Lett. 2015, 15 (11), 7748–7754. 10.1021/acs.nanolett.5b03938. 26471761

[ref36] Bacri L.; Mamad-Hemouch H.; Przybylski C.; Thiébot B.; Patriarche G.; Jarroux N.; Pelta J. Biomimetic Ion Channels Formation by Emulsion Based on Chemically Modified Cyclodextrin Nanotubes. Faraday Discuss 2018, 210 (0), 41–54. 10.1039/C8FD00030A. 29974104

[ref37] Mamad-Hemouch H.; Bacri L.; Huin C.; Przybylski C.; Thiébot B.; Patriarche G.; Jarroux N.; Pelta J. Versatile Cyclodextrin Nanotube Synthesis with Functional Anchors for Efficient Ion Channel Formation: Design, Characterization and Ion Conductance. Nanoscale 2018, 10 (32), 15303–15316. 10.1039/C8NR02623H. 30069556

[ref38] Harada A.; Li J.; Kamachi M. Synthesis of a Tubular Polymer from Threaded Cyclodextrins. Nature 1993, 364 (6437), 516–518. 10.1038/364516a0.

[ref39] Lien L.; Jaikaran D. C. J.; Zhang Z.; Woolley G. A. Photomodulated Blocking of Gramicidin Ion Channels. J. Am. Chem. Soc. 1996, 118 (48), 12222–12223. 10.1021/ja962217s.

[ref40] Jog P. V.; Gin M. S. A Light-Gated Synthetic Ion Channel. Org. Lett. 2008, 10 (17), 3693–3696. 10.1021/ol8013045. 18656946

[ref41] Zhou Y.; Chen Y.; Zhu P. P.; Si W.; Hou J. L.; Liu Y. Reversible Photo-Gated Transmembrane Channel Assembled from an Acylhydrazone-Containing Crown Ether Triad. Chem. Commun. 2017, 53 (26), 3681–3684. 10.1039/C7CC01123G. 28294246

[ref42] Muraoka T.; Umetsu K.; Tabata K. V.; Hamada T.; Noji H.; Yamashita T.; Kinbara K. Mechano-Sensitive Synthetic Ion Channels. J. Am. Chem. Soc. 2017, 139 (49), 18016–18023. 10.1021/jacs.7b09515. 29077401

[ref43] Yang R. Y.; Bao C. Y.; Lin Q. N.; Zhu L. Y. A Light-Regulated Synthetic Ion Channel Constructed by an Azobenzene Modified Hydraphile. Chin. Chem. Lett. 2015, 26 (7), 851–856. 10.1016/j.cclet.2015.05.010.

[ref44] Liu T.; Bao C.; Wang H.; Lin Y.; Jia H.; Zhu L. Light-Controlled Ion Channels Formed by Amphiphilic Small Molecules Regulate Ion Conduction via Cis-Trans Photoisomerization. Chem. Commun. 2013, 49 (87), 10311 10.1039/c3cc45618h. 24064555

[ref45] Haswell E. S.; Phillips R.; Rees D. C. Mechanosensitive Channels: What Can They Do and How Do They Do It?. Structure 2011, 19 (10), 1356–1369. 10.1016/j.str.2011.09.005. 22000509 PMC3203646

[ref46] Sakai N.; Mareda J.; Matile S. Artificial β-Barrels. Acc. Chem. Res. 2008, 41 (10), 1354–1365. 10.1021/ar700229r. 18590283

[ref47] Sakai N.; Mareda J.; Matile S. Rigid-Rod Molecules in Biomembrane Models: From Hydrogen-Bonded Chains to Synthetic Multifunctional Pores. Acc. Chem. Res. 2005, 38 (2), 79–87. 10.1021/ar0400802. 15709727

[ref48] Bhosale S.; Sisson A. L.; Talukdar P.; Fürstenberg A.; Banerji N.; Vauthey E.; Bollot G.; Mareda J.; Röger C.; Würthner F.; Sakai N.; Matile S. Photoproduction of Proton Gradients with π-Stacked Fluorophore Scaffolds in Lipid Bilayers. Science 2006, 313 (5783), 84–86. 10.1126/science.1126524. 16825567

[ref49] Talukdar P.; Bollot G.; Mareda J.; Sakai N.; Matile S. Ligand-Gated Synthetic Ion Channels. Chem. Eur. J. 2005, 11 (22), 6525–6532. 10.1002/chem.200500516. 16118825

[ref50] Talukdar P.; Bollot G.; Mareda J.; Sakai N.; Matile S. Synthetic Ion Channels with Rigid-Rod π-Stack Architecture That Open in Response to Charge-Transfer Complex Formation. J. Am. Chem. Soc. 2005, 127 (18), 6528–6529. 10.1021/ja051260p. 15869262

[ref51] Wang W.-Z. Z.; Huang L.-B. B.; Zheng S.-P. P.; Moulin E.; Gavat O.; Barboiu M.; Giuseppone N. Light-Driven Molecular Motors Boost the Selective Transport of Alkali Metal Ions through Phospholipid Bilayers. J. Am. Chem. Soc. 2021, 143 (38), 15653–15660. 10.1021/jacs.1c05750. 34520204

[ref52] Yang H.; Yi J.; Pang S.; Ye K.; Ye Z.; Duan Q.; Yan Z.; Lian C.; Yang Y.; Zhu L.; Qu D. H.; Bao C. A Light-Driven Molecular Machine Controls K+ Channel Transport and Induces Cancer Cell Apoptosis. Angew. Chem., Int. Ed. 2022, 61 (26), e202204605 10.1002/anie.202204605. 35442566

[ref53] Chen J.; Kistemaker J. C. M.; Robertus J.; Feringa B. L. Molecular Stirrers in Action. J. Am. Chem. Soc. 2014, 136 (42), 14924–14932. 10.1021/ja507711h. 25254645

[ref54] Läuger P. Mechanisms of Biological Ion Transport — Carriers, Channels, and Pumps in Artificial Lipid Membranes. Angew. Chem., Int. Ed. 1985, 24, 905–923. 10.1002/anie.198509051.

[ref55] Spooner M. J.; Gale P. A. Anion Transport across Varying Lipid Membranes - the Effect of Lipophilicity. Chem. Commun. 2015, 51 (23), 4883–4886. 10.1039/C5CC00823A. 25704626

[ref56] McNally B. A.; Koulov A. V.; Lambert T. N.; Smith B. D.; Joos J. B.; Sisson A. L.; Clare J. P.; Sgarlata V.; Judd L. W.; Magro G.; Davis A. P. Structure-Activity Relationships in Cholapod Anion Carriers: Enhanced Transmembrane Chloride Transport through Substituent Tuning. Chem. Eur. J. 2008, 14 (31), 9599–9606. 10.1002/chem.200801163. 18773409 PMC2849112

[ref57] Haynes C. J. E.; Moore S. J.; Hiscock J. R.; Marques I.; Costa P. J.; Félix V.; Gale P. A. Tunable Transmembrane Chloride Transport by Bis-Indolylureas. Chem. Sci. 2012, 3 (5), 1436–1444. 10.1039/c2sc20041d.

[ref58] Johnson T. G.; Docker A.; Sadeghi-Kelishadi A.; Langton M. J. Halogen Bonding Relay and Mobile Anion Transporters with Kinetically Controlled Chloride Selectivity. Chem. Sci. 2023, 14 (19), 5006–5013. 10.1039/D3SC01170D. 37206385 PMC10189858

[ref59] Beeren S. R.; McTernan C. T.; Schaufelberger F. The Mechanical Bond in Biological Systems. Chem. 2023, 9 (6), 1378–1412. 10.1016/j.chempr.2023.03.030.

[ref60] Pairault N.; Barat R.; Tranoy-Opalinski I.; Renoux B.; Thomas M.; Papot S. Rotaxane-Based Architectures for Biological Applications. Comptes Rendus Chimie 2016, 19 (1–2), 103–112. 10.1016/j.crci.2015.05.012.

[ref61] Riebe J.; Niemeyer J. Mechanically Interlocked Molecules for Biomedical Applications. Eur. J. Org. Chem. 2021, 2021 (37), 5106–5116. 10.1002/ejoc.202100749.

[ref62] Dvornikovs V.; House B. E.; Kaetzel M.; Dedman J. R.; Smithrud D. B. Host-[2]Rotaxanes as Cellular Transport Agents. J. Am. Chem. Soc. 2003, 125 (27), 8290–8301. 10.1021/ja034918c. 12837101

[ref63] Bao X.; Isaacsohn I.; Drew A. F.; Smithrud D. B. Determining the Intracellular Transport Mechanism of a Cleft-[2]Rotaxane. J. Am. Chem. Soc. 2006, 128 (37), 12229–12238. 10.1021/ja063667f. 16967974

[ref64] Wang X.; Bao X.; McFarland-Mancini M.; Isaacsohn I.; Drew A. F.; Smithrud D. B. Investigation of the Intracellular Delivery of Fluoresceinated Peptides by a Host-[2]Rotaxane. J. Am. Chem. Soc. 2007, 129 (23), 7284–7293. 10.1021/ja067928x. 17516642

[ref65] Zhu J.; McFarland-Mancini M.; Drew A. F.; Smithrud D. B. Host-Rotaxanes with Oligomeric Axles Are Intracellular Transport Agents. Bioorg. Med. Chem. Lett. 2009, 19 (2), 520–523. 10.1016/j.bmcl.2008.11.053. 19081721

[ref66] Zhu J.; House B. E.; Fleck E.; Isaacsohn I.; Drew A. F.; Smithrud D. B. A Host-Rotaxane Derivatized with Carboxylic Acids Efficiently Delivers a Highly Cationic Fluoresceinated Peptide. Bioorg. Med. Chem. Lett. 2007, 17 (18), 5058–5062. 10.1016/j.bmcl.2007.07.013. 17656089

[ref67] Smithrud D. B.; Wang X.; Tarapore P.; Ho S. M. Crown Ether Host-Rotaxanes as Cytotoxic Agents. ACS Med. Chem. Lett. 2013, 4 (1), 27–31. 10.1021/ml3003204. 23538490 PMC3607455

[ref68] Smithrud D. B.; Powers L.; Lunn J.; Abernathy S.; Peschka M.; Ho S.-m.; Tarapore P. Ca2+ Selective Host Rotaxane Is Highly Toxic Against Prostate Cancer Cells. ACS Med. Chem. Lett. 2017, 8 (2), 163–167. 10.1021/acsmedchemlett.6b00347. 28197305 PMC5304289

[ref69] Shi J.; Xu Y.; Wang X.; Zhang L.; Zhu J.; Pang T.; Bao X. Synthesis and Evaluation of a Novel Rhodamine B Pyrene [2]Rotaxane as an Intracellular Delivery Agent for Doxorubicin. Org. Biomol. Chem. 2015, 13 (27), 7517–7529. 10.1039/C5OB00934K. 26073047

[ref70] Chhun C.; Richard-Daniel J.; Kempf J.; Schmitzer A. R. Transport of Macrocyclic Compounds across Phospholipid Bilayers by Umbrella-Rotaxanes. Org. Biomol. Chem. 2013, 11 (36), 6023–6028. 10.1039/c3ob41209a. 23903771

[ref71] Chhun C.; Schmitzer A. R. A Pseudorotaxane Umbrella Thread with Chloride Transmembrane Transport Properties. Med. Chem. Commun. 2011, 2 (10), 987–990. 10.1039/c1md00128k.

[ref72] Moon C.; Kwon Y. M.; Lee W. K.; Park Y. J.; Yang V. C. In Vitro Assessment of a Novel Polyrotaxane-Based Drug Delivery System Integrated with a Cell-Penetrating Peptide. J. Controlled Release 2007, 124 (1–2), 43–50. 10.1016/j.jconrel.2007.08.029. PMC2211426 17904680

[ref73] Tamura A.; Yui N. β-Cyclodextrin-Threaded Biocleavable Polyrotaxanes Ameliorate Impaired Autophagic Flux in Niemann-Pick Type C Disease. J. Bio. Chem. 2015, 290 (15), 9442–9454. 10.1074/jbc.M115.636803. 25713067 PMC4392250

[ref74] D’Orchymont F.; Holland J. P. Asymmetric Rotaxanes as Dual-Modality Supramolecular Imaging Agents for Targeting Cancer Biomarkers. Commun. Chem. 2023, 6, 107 10.1038/s42004-023-00906-5. 37264077 PMC10235045

[ref75] Barat R.; Legigan T.; Tranoy-Opalinski I.; Renoux B.; Peraudeau E.; Clarhaut J.; Poinot P.; Fernandes A. E.; Aucagne V.; Leigh D. A.; Papot S. A Mechanically Interlocked Molecular System Programmed for the Delivery of an Anticancer Drug. Chem. Sci. 2015, 6, 2608–2613. 10.1039/C5SC00648A. 29308165 PMC5649224

[ref76] Fernandes A.; Viterisi A.; Coutrot F.; Potok S.; Leigh D. A.; Aucagne V.; Papot S. Peptide Rotaxanes Rotaxane-Based Propeptides: Protection and Enzymatic Release of a Bioactive Pentapeptide. Angew. Chem., Int. Ed. 2009, 121, 6565–6569. 10.1002/ange.200903215. 19637268

[ref77] Kench T.; Summers P. A.; Kuimova M. K.; Lewis J. E. M.; Vilar R. Rotaxanes as Cages to Control DNA Binding, Cytotoxicity, and Cellular Uptake of a Small Molecule. Angew. Chem., Int. Ed. 2021, 60 (19), 10928–10934. 10.1002/anie.202100151. 33577711

[ref78] Min Tay H.; Johnson T. G.; Docker A.; Langton M. J.; Beer P. D. Exploiting the Catenane Mechanical Bond Effect for Selective Halide Anion Transmembrane Transport. Angew. Chem., Int. Ed. 2023, 62, e202312745 10.1002/anie.202312745. 37772928

[ref79] Astumian R. D.; Pezzato C.; Feng Y.; Qiu Y.; McGonigal P. R.; Cheng C.; Stoddart J. F. Non-Equilibrium Kinetics and Trajectory Thermodynamics of Synthetic Molecular Pumps. Materials Chemistry Frontiers. 2020, 4, 1304–1314. 10.1039/D0QM00022A.

[ref80] Harris J. D.; Moran M. J.; Aprahamian I. New Molecular Switch Architectures. Proc. Natl. Acad. Sci. 2018, 115 (38), 9414–9422. 10.1073/pnas.1714499115. 30012601 PMC6156620

[ref81] Ahmad M.; Metya S.; Das A.; Talukdar P. A Sandwich Azobenzene-Diamide Dimer for Photoregulated Chloride Transport. Chem.-Eur. J. 2020, 26 (40), 8703–8708. 10.1002/chem.202000400. 32129531

[ref82] Kerckhoffs A.; Langton M. J. Reversible Photo-Control over Transmembrane Anion Transport Using Visible-Light Responsive Supramolecular Carriers. Chem. Sci. 2020, 11 (24), 6325–6331. 10.1039/D0SC02745F. 32953027 PMC7472928

[ref83] Kerckhoffs A.; Bo Z.; Penty S. E.; Duarte F.; Langton M. J. Red-Shifted Tetra-Ortho-Halo-Azobenzenes for Photo-Regulated Transmembrane Anion Transport. Org. Biomol. Chem. 2021, 19 (41), 9058–9067. 10.1039/D1OB01457A. 34617944

[ref84] Jin T. Calixarene-Based Photoresponsive Ion Carrier for the Control of Na+ Flux across a Lipid Bilayer Membrane by Visible Light. Mater. Lett. 2007, 61 (3), 805–808. 10.1016/j.matlet.2006.05.064.

[ref85] Shinkai S.; Nakaji T.; Ogawa T.; Shigematsu K.; Manabe O. Photoresponsive Crown Ethers. 2. Photocontrol of Ion Extraction and Ion Transport by a Bis(Crown Ether) with a Butterfly-like Motion. J. Am. Chem. Soc. 1981, 103 (1), 111–115. 10.1021/ja00391a021.

[ref86] Khairutdinov R. F.; Hurst J. K. Light-Driven Transmembrane Ion Transport by Spiropyran-Crown Ether Supramolecular Assemblies. Langmuir 2004, 20 (5), 1781–1785. 10.1021/la035683l.

[ref87] Jin T. Photocontrol of Na+ Transport across a Phospholipid Bilayer Containing a Bisanthroylcalix[4]Arene Carrier. Chem. Commun. 2000, 0 (15), 1379–1380. 10.1039/b002034f.

[ref88] Choi Y. R.; Kim G. C.; Jeon H. G.; Park J.; Namkung W.; Jeong K. S. Azobenzene-Based Chloride Transporters with Light-Controllable Activities. Chem. Commun. 2014, 50 (97), 15305–15308. 10.1039/C4CC07560A. 25350406

[ref89] Ahmad M.; Mondal D.; Roy N. J.; Vijayakanth T.; Talukdar P. Reversible Stimuli-Responsive Transmembrane Ion Transport Using Phenylhydrazone-Based Photoswitches. Chem. Photo. Chem. 2022, 6 (6), e202200002 10.1002/cptc.202200002.

[ref90] Ahmad M.; Chattopadhayay S.; Mondal D.; Vijayakanth T.; Talukdar P. Stimuli-Responsive Anion Transport through Acylhydrazone-Based Synthetic Anionophores. Org. Lett. 2021, 23 (19), 7319–7324. 10.1021/acs.orglett.1c02249. 34519509

[ref91] Santacroce P. V.; Davis J. T.; Light M. E.; Gale P. A.; Iglesias-Sanchez J. C.; Prados P.; Quesada R. Conformational Control of Transmembrane Cl- Transport. J. Am. Chem. Soc. 2007, 129 (7), 1886–1887. 10.1021/ja068067v. 17253691

[ref92] Shen F. F.; Dai S. Y.; Wong N. K.; Deng S.; Wong A. S. T.; Yang D. Mediating K+/H+ Transport on Organelle Membranes to Selectively Eradicate Cancer Stem Cells with a Small Molecule. J. Am. Chem. Soc. 2020, 142 (24), 10769–10779. 10.1021/jacs.0c02134. 32441923

[ref93] Howe E. N. W.; Busschaert N.; Wu X.; Berry S. N.; Ho J.; Light M. E.; Czech D. D.; Klein H. A.; Kitchen J. A.; Gale P. A. PH-Regulated Nonelectrogenic Anion Transport by Phenylthiosemicarbazones. J. Am. Chem. Soc. 2016, 138 (26), 8301–8308. 10.1021/jacs.6b04656. 27299473

[ref94] Howe E. N. W.; Chang V. V. T.; Wu X.; Fares M.; Lewis W.; Macreadie L. K.; Gale P. A. Halide-Selective, Proton-Coupled Anion Transport by Phenylthiosemicarbazones. Biochimica et Biophysica Acta (BBA) - Biomembranes 2022, 1864 (2), 183828 10.1016/j.bbamem.2021.183828. 34861222

[ref95] Wezenberg S. J.; Chen L. J.; Bos J. E.; Feringa B. L.; Howe E. N. W.; Wu X.; Siegler M. A.; Gale P. A. Photomodulation of Transmembrane Transport and Potential by Stiff-Stilbene Based Bis(Thio)Ureas. J. Am. Chem. Soc. 2022, 144 (1), 331–338. 10.1021/jacs.1c10034. 34932344 PMC8759083

[ref96] Villarón D.; Bos J. E.; Kohl F.; Mommer S.; de Jong J.; Wezenberg S. J. Photoswitchable Bis(Amidopyrroles): Modulating Anion Transport Activity Independent of Binding Affinity. J. Org. Chem. 2023, 88 (15), 11328–11334. 10.1021/acs.joc.3c01018. 37440304 PMC10407928

[ref97] Martins J. N.; Raimundo B.; Rioboo A.; Folgar-Cameán Y.; Montenegro J.; Basílio N. Photoswitchable Calixarene Activators for Controlled Peptide Transport across Lipid Membranes. J. Am. Chem. Soc. 2023, 145 (24), 13126–13133. 10.1021/jacs.3c01829. 37289668 PMC10288505

[ref98] Shen J.; Ren C.; Zeng H. Membrane-Active Molecular Machines. Acc. Chem. Res. 2022, 55 (8), 1148–1159. 10.1021/acs.accounts.1c00804. 35345880

[ref99] Ye R.; Ren C.; Shen J.; Li N.; Chen F.; Roy A.; Zeng H. Molecular Ion Fishers as Highly Active and Exceptionally Selective K+ Transporters. J. Am. Chem. Soc. 2019, 141 (25), 9788–9792. 10.1021/jacs.9b04096. 31184884

[ref100] Ren C.; Chen F.; Ye R.; Ong Y. S.; Lu H.; Lee S. S.; Ying J. Y.; Zeng H. Molecular Swings as Highly Active Ion Transporters. Angew. Chem., Int. Ed. 2019, 58 (24), 8034–8038. 10.1002/anie.201901833. 30983075

[ref101] Li N.; Shen J.; Ang G. K.; Ye R.; Zeng H. Molecular Tetrahedrons as Selective and Efficient Ion Transporters via a Two-Station Swing-Relay Mechanism. CCS Chemistry 2021, 3 (8), 2269–2279. 10.31635/ccschem.020.202000475.

[ref102] Shen J.; Han J. J. Y.; Ye R.; Zeng H. Molecular Rotors as a Class of Generally Highly Active Ion Transporters. Sci. China Chem 2021, 64 (12), 2154–2160. 10.1007/s11426-021-1082-7.

[ref103] Miao M.; Shao X.; Cai W. Conformational Change from U-to I-Shape of Ion Transporters Facilitates K+ Transport across Lipid Bilayers. J. Phys. Chem. B 2022, 126 (7), 1520–1528. 10.1021/acs.jpcb.1c09423. 35142530

[ref104] Chen S.; Wang Y.; Nie T.; Bao C.; Wang C.; Xu T.; Lin Q.; Qu D. H.; Gong X.; Yang Y.; Zhu L.; Tian H. An Artificial Molecular Shuttle Operates in Lipid Bilayers for Ion Transport. J. Am. Chem. Soc. 2018, 140 (51), 17992–17998. 10.1021/jacs.8b09580. 30445811

[ref105] Wang C.; Wang S.; Yang H.; Xiang Y.; Wang X.; Bao C.; Zhu L.; Tian H.; Qu D. H. A Light-Operated Molecular Cable Car for Gated Ion Transport. Angew. Chem., Int. Ed. 2021, 60 (27), 14836–14840. 10.1002/anie.202102838. 33843130

[ref106] Li N.; Chen F.; Shen J.; Zhang H.; Wang T.; Ye R.; Li T.; Loh T. P.; Yang Y. Y.; Zeng H. Buckyball-Based Spherical Display of Crown Ethers for de Novo Custom Design of Ion Transport Selectivity. J. Am. Chem. Soc. 2020, 142 (50), 21082–21090. 10.1021/jacs.0c09655. 33274928

[ref107] Otis F.; Racine-Berthiaume C.; Voyer N. How Far Can a Sodium Ion Travel within a Lipid Bilayer?. J. Am. Chem. Soc. 2011, 133 (17), 6481–6483. 10.1021/ja110336s. 21384853

[ref108] Zhang H.; Guo Y.; Chipot C.; Cai W.; Shao X. Nanomachine-Assisted Ion Transport across Membranes: From Mechanism to Rational Design and Applications. J. Phys. Chem. Lett. 2021, 12 (13), 3281–3287. 10.1021/acs.jpclett.1c00525. 33764777

[ref109] Pang S.; Sun X.; Yan Z.; Wang C.; Ye K.; Ma S.; Zhu L.; Bao C. A Rigid-Axle-Based Molecular Rotaxane Channel Facilitates K+/Cl- Co-Transport across a Lipid Membrane. Chem. Commun. 2023, 59 (26), 3866–3869. 10.1039/D3CC00811H. 36897090

[ref110] Ye K.; Zhang Z.; Yan Z.; Pang S.; Yang H.; Sun X.; Liu C.; Zhu L.; Lian C.; Bao C. Molecular Rotaxane Shuttle-Relay Accelerates K+/Cl- Symport across a Lipid Membrane. Sci China Chem 2023, 66 (8), 2300–2308. 10.1007/s11426-023-1614-7.

[ref111] McNally B. A.; O’Neil E. J.; Nguyen A.; Smith B. D. Membrane Transporters for Anions That Use a Relay Mechanism. J. Am. Chem. Soc. 2008, 130 (51), 17274–17275. 10.1021/ja8082363. 19035637 PMC2814302

[ref112] Johnson T. G.; Docker A.; Sadeghi-Kelishadi A.; Langton M. J. Halogen Bonding Relay and Mobile Anion Transporters with Kinetically Controlled Chloride Selectivity. Chem. Sci. 2023, 14 (19), 5006–5013. 10.1039/D3SC01170D. 37206385 PMC10189858

[ref113] Weber M. E.; Schlesinger P. H.; Gokel G. W. Dynamic Assessment of Bilayer Thickness by Varying Phospholipid and Hydraphile Synthetic Channel Chain Lengths. J. Am. Chem. Soc. 2005, 127 (2), 636–642. 10.1021/ja044936+. 15643888 PMC2615579

[ref114] Benz R.; Stark G.; Janko K.; Läuger P. Valinomycin-Mediated Ion Transport through Neutral Lipid Membranes: Influence of Hydrocarbon Chain Length and Temperature. J. Membr. Biol. 1973, 14 (1), 339–364. 10.1007/BF01868084. 4781449

[ref115] Benz R.; Fröhlich O.; Läuger P. Influence of Membrane Structure on the Kinetics of Carrier-Mediated Ion Transport through Lipid Bilayers. Biochimica et Biophysica Acta (BBA) - Biomembranes 1977, 464 (3), 465–481. 10.1016/0005-2736(77)90023-2. 836821

[ref116] Johnson T. G.; Sadeghi-Kelishadi A.; Langton M. J. A Photo-Responsive Transmembrane Anion Transporter Relay. J. Am. Chem. Soc. 2022, 144 (23), 10455–10461. 10.1021/jacs.2c02612. 35652660 PMC9204766

[ref117] Bléger D.; Schwarz J.; Brouwer A. M.; Hecht S. O -Fluoroazobenzenes as Readily Synthesized Photoswitches Offering Nearly Quantitative Two-Way Isomerization with Visible Light. J. Am. Chem. Soc. 2012, 134 (51), 20597–20600. 10.1021/ja310323y. 23236950

[ref118] Fitzmaurice O.; Bartkowski M.; Giordani S. Molecular Switches—Tools for Imparting Control in Drug Delivery Systems. Frontiers in Chemistry. 2022, 10, 859450 10.3389/fchem.2022.859450. 35433638 PMC9008311

[ref119] Weil T.; Groß R.; Röcker A.; Bravo-Rodriguez K.; Heid C.; Sowislok A.; Le M.-H.; Erwin N.; Dwivedi M.; Bart S. M.; Bates P.; Wettstein L.; Müller J. A.; Harms M.; Sparrer K.; Ruiz-Blanco Y. B.; Stürzel C. M.; von Einem J.; Lippold S.; Read C.; Walther P.; Hebel M.; Kreppel F.; Klärner F.-G.; Bitan G.; Ehrmann M.; Weil T.; Winter R.; Schrader T.; Shorter J.; Sanchez-Garcia E.; Münch J. Supramolecular Mechanism of Viral Envelope Disruption by Molecular Tweezers. J. Am. Chem. Soc. 2020, 142 (40), 17024–17038. 10.1021/jacs.0c06400. 32926779 PMC7523239

[ref120] Shahpasand-Kroner H.; Siddique I.; Malik R.; Linares G. R.; Ivanova M. I.; Ichida J.; Weil T.; Münch J.; Sanchez-Garcia E.; Klärner F. G.; Schrader T.; Bitan G. Molecular Tweezers: Supramolecular Hosts with Broad-Spectrum Biological Applications. Pharmacol. Rev. 2023, 75 (2), 263–308. 10.1124/pharmrev.122.000654. 36549866 PMC9976797

[ref121] Morstein J.; Impastato A. C.; Trauner D. Photoswitchable Lipids. Chem. Bio. Chem. 2021, 22, 73–83. 10.1002/cbic.202000449. 32790211

[ref122] Liu D.; Wang S.; Xu S.; Liu H. Photocontrollable Intermittent Release of Doxorubicin Hydrochloride from Liposomes Embedded by Azobenzene-Contained Glycolipid. Langmuir 2017, 33 (4), 1004–1012. 10.1021/acs.langmuir.6b03051. 27668306

[ref123] Peer D.; Karp J. M.; Hong S.; Farokhzad O. C.; Margalit R.; Langer R. Nanocarriers as an Emerging Platform for Cancer Therapy. Nature Nanotechnology 2007, 2 (12), 751–760. 10.1038/nnano.2007.387. 18654426

[ref124] Hu J.; Wu D.; Pan Q.; Li H.; Zhang J.; Geng F. Recent Development of Photoresponsive Liposomes Based on Organic Photosensitizers, Au Nanoparticles, and Azobenzene Derivatives for Nanomedicine. ACS Appl. Nano. Mater. 2022, 5 (10), 14171–14190. 10.1021/acsanm.2c03167.

[ref125] Chander N.; Morstein J.; Bolten J. S.; Shemet A.; Cullis P. R.; Trauner D.; Witzigmann D. Optimized Photoactivatable Lipid Nanoparticles Enable Red Light Triggered Drug Release. Small 2021, 17 (21), 2008198 10.1002/smll.202008198. 33880882

[ref126] Lei Y.; Hurst J. K. Photoregulated Potassium Ion Permeation through Dihexadecyl Phosphate Bilayers Containing Azobenzene and Stilbene Surfactants. Langmuir 1999, 15 (10), 3424–3429. 10.1021/la981223u.

[ref127] Moss R. A.; Jiang W. Cis/Trans Isomerization in Azobenzene-Chain Liposomes. Langmuir 1995, 11, 4217–4221. 10.1021/la00011a010.

[ref128] Cui Z.-K.; Phoeung T.; Rousseau P.-A.; Rydzek G.; Zhang Q.; Bazuin C. G.; Lafleur M. Nonphospholipid Fluid Liposomes with Switchable Photocontrolled Release. Langmuir 2014, 30 (36), 10818–10825. 10.1021/la502131h. 25149436

[ref129] Uchida N.; Ryu Y.; Takagi Y.; Yoshizawa K.; Suzuki K.; Anraku Y.; Ajioka I.; Shimokawa N.; Takagi M.; Hoshino N.; Akutagawa T.; Matsubara T.; Sato T.; Higuchi Y.; Ito H.; Morita M.; Muraoka T. Endocytosis-Like Vesicle Fission Mediated by a Membrane-Expanding Molecular Machine Enables Virus Encapsulation for In Vivo Delivery. J. Am. Chem. Soc. 2023, 145 (11), 6210–6220. 10.1021/jacs.2c12348. 36853954 PMC10037323

[ref130] Pritzl S. D.; Konrad D. B.; Ober M. F.; Richter A. F.; Frank J. A.; Nickel B.; Trauner D.; Lohmüller T. Optical Membrane Control with Red Light Enabled by Red-Shifted Photolipids. Langmuir 2022, 38 (1), 385–393. 10.1021/acs.langmuir.1c02745. 34969246

[ref131] Rojas-Gutierrez P. A.; Bhuckory S.; Mingoes C.; Hildebrandt N.; Dewolf C.; Capobianco J. A. A Route to Triggered Delivery via Photocontrol of Lipid Bilayer Properties Using Lanthanide Upconversion Nanoparticles. ACS Appl. Nano. Mater. 2018, 1 (9), 5345–5354. 10.1021/acsanm.8b01396.

[ref132] Yao C.; Wang P.; Li X.; Hu X.; Hou J.; Wang L.; Zhang F. Near-Infrared-Triggered Azobenzene-Liposome/Upconversion Nanoparticle Hybrid Vesicles for Remotely Controlled Drug Delivery to Overcome Cancer Multidrug Resistance. Adv. Mater. 2016, 28 (42), 9341–9348. 10.1002/adma.201503799. 27578301

[ref133] Guesdon-Vennerie A.; Couvreur P.; Ali F.; Pouzoulet F.; Roulin C.; Martínez-Rovira I.; Bernadat G.; Legrand F. X.; Bourgaux C.; Mazars C. L.; Marco S.; Trépout S.; Mura S.; Mériaux S.; Bort G. Breaking Photoswitch Activation Depth Limit Using Ionising Radiation Stimuli Adapted to Clinical Application. Nat. Commun. 2022, 13 (1), 4102 10.1038/s41467-022-30917-0. 35835744 PMC9283480

[ref134] Brazdova B.; Zhang N.; Samoshin V. V.; Guo X. Trans-2-Aminocyclohexanol as a PH-Sensitive Conformational Switch in Lipid Amphiphiles. Chem. Commun. 2008, 39, 4774–4776. 10.1039/b807704e. 18830489

[ref135] Viricel W.; Mbarek A.; Leblond J. Switchable Lipids: Conformational Change for Fast PH-Triggered Cytoplasmic Delivery. Angew. Chem., Int. Ed. 2015, 54 (43), 12743–12747. 10.1002/anie.201504661. 26189870

[ref136] Viricel W.; Poirier S.; Mbarek A.; Derbali R. M.; Mayer G.; Leblond J. Cationic Switchable Lipids: PH-Triggered Molecular Switch for SiRNA Delivery. Nanoscale 2017, 9 (1), 31–36. 10.1039/C6NR06701H. 27906384

[ref137] Hu B.; Zhong L.; Weng Y.; Peng L.; Huang Y.; Zhao Y.; Liang X. J. Therapeutic SiRNA: State of the Art. Signal Transduction and Targeted Therapy 2020, 5 (1), 1–25. 10.1038/s41392-020-0207-x. 32561705 PMC7305320

[ref138] Chen J.; Leung F. K. C.; Stuart M. C. A.; Kajitani T.; Fukushima T.; Van Der Giessen E.; Feringa B. L. Artificial Muscle-like Function from Hierarchical Supramolecular Assembly of Photoresponsive Molecular Motors. Nat. Chem. 2018, 10 (2), 132–138. 10.1038/nchem.2887. 29359757

[ref139] Van Dijken D. J.; Chen J.; Stuart M. C. A.; Hou L.; Feringa B. L. Amphiphilic Molecular Motors for Responsive Aggregation in Water. J. Am. Chem. Soc. 2016, 138 (2), 660–669. 10.1021/jacs.5b11318. 26700073

[ref140] Yu J. J.; Cao Z. Q.; Zhang Q.; Yang S.; Qu D. H.; Tian H. Photo-Powered Stretchable Nano-Containers Based on Well-Defined Vesicles Formed by an Overcrowded Alkene Switch. Chem. Commun. 2016, 52 (81), 12056–12059. 10.1039/C6CC06458B. 27711316

[ref141] García-López V.; Liu D.; Tour J. M. Light-Activated Organic Molecular Motors and Their Applications. Chem. Rev. 2020, 120 (1), 79–124. 10.1021/acs.chemrev.9b00221. 31849216

[ref142] García-López V.; Chen F.; Nilewski L. G.; Duret G.; Aliyan A.; Kolomeisky A. B.; Robinson J. T.; Wang G.; Pal R.; Tour J. M. Molecular Machines Open Cell Membranes. Nature 2017, 548 (7669), 567–572. 10.1038/nature23657. 28858304

[ref143] Firsov A. M.; Pfeffermann J.; Benditkis A. S.; Rokitskaya T. I.; Kozlov A. S.; Kotova E. A.; Krasnovsky A. A.; Pohl P.; Antonenko Y. N. Photodynamic Activity Rather than Drilling Causes Membrane Damage by a Light-Powered Molecular Nanomotor. J. Photochem. Photobiol. B 2023, 239, 112633 10.1016/j.jphotobiol.2022.112633. 36608401

[ref144] Santos A. L.; van Venrooy A.; Reed A. K.; Wyderka A. M.; García-López V.; Alemany L. B.; Oliver A.; Tegos G. P.; Tour J. M. Hemithioindigo-Based Visible Light-Activated Molecular Machines Kill Bacteria by Oxidative Damage. Advanced Science 2022, 9 (30), 2203242 10.1002/advs.202203242. 36002317 PMC9596824

[ref145] Guinart A.; Korpidou M.; Doellerer D.; Pacella G.; Stuart M. C. A.; Dinu I. A.; Portale G.; Palivan C.; Feringa B. L. Synthetic Molecular Motor Activates Drug Delivery from Polymersomes. Proc. Natl. Acad. Sci. 2023, 120 (27), e2301279120 10.1073/pnas.2301279120. 37364098 PMC10319042

[ref146] Lubbe A. S.; Böhmer C.; Tosi F.; Szymanski W.; Feringa B. L. Molecular Motors in Aqueous Environment. J. Org. Chem. 2018, 83 (18), 11008–11018. 10.1021/acs.joc.8b01627. 30130964 PMC6154213

[ref147] Chen S.; Leung F. K.-C.; Stuart M. C. A.; Wang C.; Feringa B. L. Dynamic Assemblies of Molecular Motor Amphiphiles Control Macroscopic Foam Properties. J. Am. Chem. Soc. 2020, 142 (22), 10163–10172. 10.1021/jacs.0c03153. 32379449 PMC7273467

[ref148] Ribovski L.; Zhou Q.; Chen J.; Feringa B. L.; Van Rijn P.; Zuhorn I. S. Light-Induced Molecular Rotation Triggers on-Demand Release from Liposomes. Chem. Commun. 2020, 56 (62), 8774–8777. 10.1039/D0CC02499F. 32618300

[ref149] Galbadage T.; Liu D.; Alemany L. B.; Pal R.; Tour J. M.; Gunasekera R. S.; Cirillo J. D. Molecular Nanomachines Disrupt Bacterial Cell Wall, Increasing Sensitivity of Extensively Drug-Resistant Klebsiella Pneumoniae to Meropenem. ACS Nano 2019, 13 (12), 14377–14387. 10.1021/acsnano.9b07836. 31815423 PMC6933815

[ref150] MacDonald T. S. C.; Price W. S.; Astumian R. D.; Beves J. E. Enhanced Diffusion of Molecular Catalysts Is Due to Convection. Angew. Chem., Int. Ed. 2019, 58 (52), 18864–18867. 10.1002/anie.201910968. 31657088

[ref151] Progatzky F.; Dallman M. J.; Lo Celso C. From Seeing to Believing: Labelling Strategies for in Vivo Cell-Tracking Experiments. Interface Focus 2013, 3 (3), 20130001 10.1098/rsfs.2013.0001. 23853708 PMC3638420

[ref152] Li H.; Valkenier H.; Judd L. W.; Brotherhood P. R.; Hussain S.; Cooper J. A.; Jurček O.; Sparkes H. A.; Sheppard D. N.; Davis A. P. Efficient, Non-Toxic Anion Transport by Synthetic Carriers in Cells and Epithelia. Nat. Chem. 2016, 8 (1), 24–32. 10.1038/nchem.2384. 26673261

[ref153] Mora N. L.; Bahreman A.; Valkenier H.; Li H.; Sharp T. H.; Sheppard D. N.; Davis A. P.; Kros A. Targeted Anion Transporter Delivery by Coiled-Coil Driven Membrane Fusion. Chem. Sci. 2016, 7 (3), 1768–1772. 10.1039/C5SC04282H. 28936326 PMC5592372

[ref154] Grant B. D.; Donaldson J. G. Pathways and Mechanisms of Endocytic Recycling. Nat. Rev. Mol. Cell Biol. 2009, 10 (9), 597–608. 10.1038/nrm2755. 19696797 PMC3038567

[ref155] Ko S. K.; Kim S. K.; Share A.; Lynch V. M.; Park J.; Namkung W.; Van Rossom W.; Busschaert N.; Gale P. A.; Sessler J. L.; Shin I. Synthetic Ion Transporters Can Induce Apoptosis by Facilitating Chloride Anion Transport into Cells. Nat. Chem. 2014, 6 (10), 885–892. 10.1038/nchem.2021. 25242483

[ref156] Gartland S. A.; Johnson T. G.; Walkley E.; Langton M. J. Inter-Vesicle Signal Transduction Using a Photo-Responsive Zinc Ionophore. Angew. Chem., Int. Ed. 2023, 62 (38), e202309080 10.1002/anie.202309080. 37497854

[ref157] De Poli M.; Zawodny W.; Quinonero O.; Lorch M.; Webb S. J.; Clayden J. Conformational Photoswitching of a Synthetic Peptide Foldamer Bound within a Phospholipid Bilayer. Science 2016, 352 (6285), 575–580. 10.1126/science.aad8352. 27033546

[ref158] Langton M. J.; Keymeulen F.; Ciaccia M.; Williams N. H.; Hunter C. A. Controlled Membrane Translocation Provides a Mechanism for Signal Transduction and Amplification. Nat. Chem. 2017, 9 (5), 426–430. 10.1038/nchem.2678. 28430205

[ref159] Pang S.; Liu J.; Li T.; Ye K.; Yan Z.; Zhao L.; Bao C. Folding and Unfolding of a Fully Synthetic Transmembrane Receptor for ON/OFF Signal Transduction. J. Am. Chem. Soc. 2023, 145 (38), 20761–20766. 10.1021/jacs.3c07814. 37699413

[ref160] Hansen M.; Li F.; Sun L.; König B. Photocatalytic Water Oxidation at Soft Interfaces. Chem. Sci. 2014, 5 (7), 2683–2687. 10.1039/C4SC01018C.

[ref161] Song H.; Amati A.; Pannwitz A.; Bonnet S.; Hammarström L. Mechanistic Insights into the Charge Transfer Dynamics of Photocatalytic Water Oxidation at the Lipid Bilayer-Water Interface. J. Am. Chem. Soc. 2022, 144 (42), 19353–19364. 10.1021/jacs.2c06842. 36250745 PMC9619399

[ref162] Deng Y.; Wu T.; Chen X.; Chen Y.; Fei Y.; Liu Y.; Chen Z.; Xing H.; Bai Y. A Membrane-Embedded Macromolecular Catalyst with Substrate Selectivity in Live Cells. J. Am. Chem. Soc. 2023, 145 (2), 1262–1272. 10.1021/jacs.2c11168. 36525295

[ref163] Frawley A. T.; Wycisk V.; Xiong Y.; Galiani S.; Sezgin E.; Urbancic I.; Vargas Jentzsch A.; Leslie K. G.; Eggeling C.; Anderson H. L. Super-Resolution RESOLFT Microscopy of Lipid Bilayers Using a Fluorophore-Switch Dyad. Chem. Sci. 2020, 11, 8955–8960. 10.1039/D0SC02447C. 34123149 PMC8163400

[ref164] Chen X. X.; Bayard F.; Gonzalez-Sanchis N.; Pamungkas K. K. P.; Sakai N.; Matile S. Fluorescent Flippers: Small-Molecule Probes to Image Membrane Tension in Living Systems. Angew. Chem., Int. Ed. 2023, 62 (20), e202217868 10.1002/anie.202217868. 36734976

[ref165] López-Andarias J.; Eblighatian K.; Pasquer Q. T. L.; Assies L.; Sakai N.; Hoogendoorn S.; Matile S. Photocleavable Fluorescent Membrane Tension Probes: Fast Release with Spatiotemporal Control in Inner Leaflets of Plasma Membrane, Nuclear Envelope, and Secretory Pathway. Angew. Chem., Int. Ed. 2022, 61, e202113163 10.1002/anie.202113163. PMC9299180 34734671

[ref166] Goujon A.; Colom A.; Straková K.; Mercier V.; Mahecic D.; Manley S.; Sakai N.; Roux A.; Matile S. Mechanosensitive Fluorescent Probes to Image Membrane Tension in Mitochondria, Endoplasmic Reticulum, and Lysosomes. J. Am. Chem. Soc. 2019, 141 (8), 3380–3384. 10.1021/jacs.8b13189. 30744381

[ref167] Colom A.; Derivery E.; Soleimanpour S.; Tomba C.; Molin M. D.; Sakai N.; González-Gaitán M.; Matile S.; Roux A. A Fluorescent Membrane Tension Probe. Nat. Chem. 2018, 10 (11), 1118–1125. 10.1038/s41557-018-0127-3. 30150727 PMC6197433

[ref168] Biagini C.; Di Stefano S. Abiotic Chemical Fuels for the Operation of Molecular Machines. Angew. Chem., Int. Ed. 2020, 59 (22), 8344–8354. 10.1002/anie.201912659. 31898850

[ref169] Borsley S.; Leigh D. A.; Roberts B. M. W. Chemical Fuels for Molecular Machinery. Nat. Chem. 2022, 14 (7), 728–738. 10.1038/s41557-022-00970-9. 35778564

[ref170] Steinberg-Yfrach G.; Rigaud J. L.; Durantini E. N.; Moore A. L.; Gust D.; Moore T. A. Light-Driven Production of ATP Catalysed by F0F1-ATP Synthase in an Artificial Photosynthetic Membrane. Nature 1998, 392 (6675), 479–482. 10.1038/33116. 9548252

[ref171] Chen S.; Yang L.; Leung F. K. C.; Kajitani T.; Stuart M. C. A.; Fukushima T.; Van Rijn P.; Feringa B. L. Photoactuating Artificial Muscles of Motor Amphiphiles as an Extracellular Matrix Mimetic Scaffold for Mesenchymal Stem Cells. J. Am. Chem. Soc. 2022, 144 (8), 3543–3553. 10.1021/jacs.1c12318. 35171583 PMC8895399

